# Nanocellulose-Based Materials for Water Treatment: Adsorption, Photocatalytic Degradation, Disinfection, Antifouling, and Nanofiltration

**DOI:** 10.3390/nano11113008

**Published:** 2021-11-09

**Authors:** Ahmed Salama, Ragab Abouzeid, Wei Sun Leong, Jaison Jeevanandam, Pieter Samyn, Alain Dufresne, Mikhael Bechelany, Ahmed Barhoum

**Affiliations:** 1 Cellulose and Paper Department, National Research Centre, 33 El-Bohouth St., Dokki, Giza 12622, Egypt; Ahmed_nigm78@yahoo.com (A.S.); r_abouzeid2002@yahoo.com (R.A.); 2 University of Grenoble Alpes, CNRS, Grenoble INP, LGP2, F-38000 Grenoble, France; alain.dufresne@pagora.grenoble-inp.fr; 3 Department of Materials Science and Engineering, National University of Singapore, Singapore 117575, Singapore; weisun@u.nus.edu; 4 CQM—Centro de Química da Madeira, MMRG, Campus da Penteada, Universidade da Madeira, 9020-105 Funchal, Portugal; jaison.jeevanandam@staff.uma.pt; 5 Institute for Materials Research (MO-IMOMEC), Applied and Analytical Chemistry, University of Hasselt, B-3590 Diepenbeek, Belgium; pieter.samyn@uhasselt.be; 6 Institut Européen des Membranes, IEM, UMR 5635, Univ Montpellier, CNRS, ENSCM, 34090 Montpellier, France; 7 NanoStruc Research Group, Chemistry Department, Faculty of Science, Helwan University, Cairo, Helwan 11795, Egypt; 8 School of Chemical Sciences, Dublin City University, Dublin 9, D09 Y074 Dublin, Ireland

**Keywords:** nanoparticles, nanocrystals, nanowhiskers, nanofibers, hydrogels, bacterial cellulose, surface functionalization, membranes filtration

## Abstract

Nanocelluloses are promising bio-nano-materials for use as water treatment materials in environmental protection and remediation. Over the past decades, they have been integrated via novel nanoengineering approaches for water treatment processes. This review aims at giving an overview of nanocellulose requirements concerning emerging nanotechnologies of waster treatments and purification, i.e., adsorption, absorption, flocculation, photocatalytic degradation, disinfection, antifouling, ultrafiltration, nanofiltration, and reverse osmosis. Firstly, the nanocellulose synthesis methods (mechanical, physical, chemical, and biological), unique properties (sizes, geometries, and surface chemistry) were presented and their use for capturing and removal of wastewater pollutants was explained. Secondly, different chemical modification approaches surface functionalization (with functional groups, polymers, and nanoparticles) for enhancing the surface chemistry of the nanocellulose for enabling the effective removal of specific pollutants (suspended particles, microorganisms, hazardous metals ions, organic dyes, drugs, pesticides fertilizers, and oils) were highlighted. Thirdly, new fabrication approaches (solution casting, thermal treatment, electrospinning, 3D printing) that integrated nanocelluloses (spherical nanoparticles, nanowhiskers, nanofibers) to produce water treatment materials (individual composite nanoparticles, hydrogels, aerogels, sponges, membranes, and nanopapers) were covered. Finally, the major challenges and future perspectives concerning the applications of nanocellulose based materials in water treatment and purification were highlighted.

## 1. Introduction

Nowadays, treatment and purification of sanitation and industrial water are very critical to the survival of people and the planet. About 97.5% of the total available water on earth is salty which is not suitable for use [1]. The remaining water is 2.5% of fresh water, only ~1%, of this fresh water is available for human consumption. Of the total wastewaters produced from human activities, 10–20% of all the wastewater is treated and reused. Therefore, billions of people around the world lack access to safe drinking water and sanitation [1]. The freshwater is contaminated due to human activities and consequently is enriched in foreign and potentially dangerous species. These pollutants can be classified into four categories: microorganisms (virus, bacteria, fungi), inorganic compounds (heavy metals, and radioactive materials), organic compounds (soaps, drugs, fertilizers, pesticides, and oils) [2]. They are all harmful to human health and the environment, induce changes in natural aqueous habitats and organisms, and affect the water quality and biodiversity. However, due to the complexity of wastewater types and the huge diversity of pollutants, specific water treatment strategies have been developed and experts are required to select the most appropriate filtering techniques and membrane materials [3].

Nanocelluloses have emerged as an alternative to conventional wastewater treatment materials. It can be produced from different sources, particularly soft and hardwood species, phloem fibers (flax, hemp, jute, ramie), grasses (bagasse, bamboo), and also bacteria, fungi, algae, and marine invertebrates [4,5,6,7]. Due to its intrinsic features, nanocelluloses based wastewater treatment materials are recommended candidates for industrial water treatment systems [8]. They can serve this goal by providing highly efficient materials for wastewater treatment owing to their high aspect ratio, surface area, surface charge, and mechanical strength. The performance of nanocelluloses based wastewater treatment materials (e.g., adsorbents, membranes, flocculants, photocatalysts, and disinfectants) has to fulfill diverse functions. However, nanocelluloses functions must be provided with an optimum balance between porosity, high permeability, selective binding, durability, and specific filtration mechanisms (i.e., size exclusion, ion exchange, adsorption). Moreover, the size, morphology, and surface chemistry of nanocelluloses need to be controlled for simultaneously tuning the affinity towards specific pollutants and avoiding microbial growth and fouling [3,9].

This review provides an overview of types, classifications, and unique properties of nanocelluloses as well as the recent methods for nanocellulose synthesis and control of their sizes, aspect ratio of a geometric shape, and pathways to rationalize their surface properties for filtration via size exclusion, absorption/adsorption, flocculation, photocatalytic degradation, disinfection, and antifouling. Intrinsic adsorption properties of nanocellulose [10], and filtration membranes [11,12,13] are highlighted in previous reviews for wastewater treatment [14]. In contrast with previously published reviews, this multidisciplinary article offers an updated and critical assessment of recent findings on nanocellulose and cellulose nanocomposite research with a focus on anti-microbial activity, photocatalytic degradation, disinfection, and antifouling, ultrafiltration, nanofiltration, and reverse osmosis to water engineers and experts in chemistry or materials science. The review discusses different chemical modification approaches for enhancing the surface chemistry of the nanocellulose based materials towards the effective removal of specific pollutants such as hazardous metal ions, organic dyes, drugs, pesticides, fertilizers, and oils. 

## 2. Production, Morphologies, and Unique Properties of Nanocelluloses 

**Figure 1:**
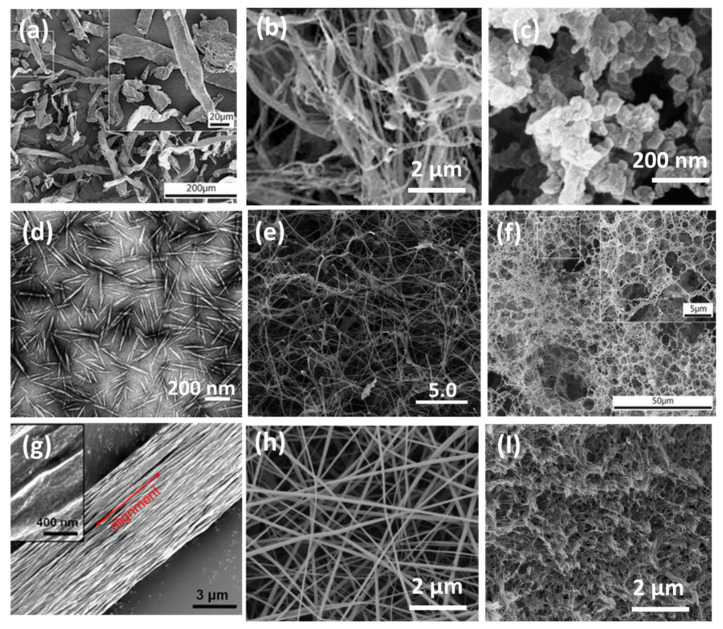
Electron microscope micrographs of different types of cellulose nanomaterials: (**a**) cellulose microfibers (CMFs) [21], © Springer Nature, 2021; (**b**) Cellulose fibers (CNFs) [22], © Hindawi, 2017; (**c**) Spherical cellulose nanoparticles (SCNPs) [23], © Elsevier, 2007; (**d**) Cellulose nanocrystals (CNCs) [24], © MDPI, 2008; (**e**) cellulose nanofibers (CNFs) [25], © Royal Society of Chemistry, 2009; (**f**) bacterial cellulose (BC); (**g**) cellulose nanofibers assembled into a material stronger than spider silk [26], © American Chemical Society, 2018; (**h**) Electrospun cellulose nanofibers (CNFs) [27], © Elsevier, 2014; (**i**) Cellulose aerogels [28], © MDPI, 2018.

The industrial-scale production and market of nanocellulose are rapidly expanding worldwide as they are environmentally friendly, non-toxic, sustainable, low-cost, and highly efficient materials for a wide variety of applications, including water treatment. At present, different forms of nanocellulose are available on the market, which is forecasted to achieve USD 783 Million by 2025 according to Markets and Markets. The global market size of nanocelluloses is expected to grow at 21.4% of a compound annual growth rate from 2020 to 2026 [5]. The main sources of nanocellulose are plants, whereas bacteria and tunicates (sea animals) are currently less used to obtain nanocelluloses [15]. To date, researchers have developed several nanofabrication techniques for the production of nanocelluloses: (1) Mechanical methods such as grinding, homogenization, refining, aqueous counter collision, cryo-crushing are normally used to disrupt the cellulose microfibers from plant materials down to submicron or nanoscale dimensions [16]. Typically, the high energy input and the microfibers suspensions with extremely high viscosity must be optimized to high yield nanocelluloses at low cost. (2) physical techniques such as steam explosion, wet spinning, dry spinning, melt spinning, or electrospinning have been used to produce electrospun cellulose acetate nanofiber (100 to 1000 nm in width and over 1 μm in length) [16,17]. Cellulose aerogels (CAG) have been fabricated by the dissolution of nanocelluloses in a favorable solvent, followed by gelation, cross-linking, solvent exchange, and drying. (3) Chemical treatment of lignocellulose materials including alkali treatment, acid hydrolysis, sulfonation, and ionic liquid approaches with selected chemicals used to provide nanocelluloses with specific functionalities and high purity [15,18,19]. (4) biological treatment such as enzymatic hydrolysis has been applied before mechanical cellulose fragmentation to reduce chemical waste and energy consumption [5,20]. Figure 1 and Table 1 summarizes the classification of the nanocelluloses according to their length, diameter, aspect ratio, and composition.

Characteristics of nanocelluloses (size, morphology, aspect ratio, surface charge) can be modulated by selecting specific raw materials, fabrication techniques, and processing parameters. The favorable characteristics of nanocellulose for employment in wastewater treatment are justified through their unique physicochemical properties (Table 2), i.e., high surface area, high aspect ratio, high mechanical stiffness, high crystalline degree, susceptible to surface functionalization, stability in water, and high surface tension. Besides these characteristics, enhanced rheological properties, alignment, and orientation, biodegradability, biocompatibility, renewable and abundancy, chemical inertness, low coefficient of thermal expansion, low density and dimensional stability are particularly interesting for water treatment applications. The nanocelluloses presents ultra-fine pore size, high surface area, and high amount of hydroxyl group [33]. The hydroxyl groups are negatively charged and exhibit electrostatic attraction toward cationic wastewater pollutants, such as heavy metals and cationic dyes, but they can also be chemically modified to improve the nanocellulose affinity for anionic and non-ionic pollutants. Interactions of water pollutants with nanocellulose surface can be further tuned through surface grafting (carboxylic, sulfate, phosphate, ester, salcilate groups) [17] or decoration with inorganic nanomaterials (metals, metal oxides, clay [34], zeolites [35], carbon nanomaterials (graphene, carbon nanotubes, active carbon [36] or polymers (ion exchange resins [37] chitosan [38,39,40] alginate [41], and lignin [42]. However, certain current drawbacks hinder the further expansion of the nanocellulose utilization as mainly related to their processing conditions:

(1) High dispersion stability of nanocelluloses in aqueous solutions can be achieved through sulfuric acid hydrolysis introducing negatively charged sulfate groups. However, this high dispersion stability of individual nanocellulose particles makes their separation from the water system difficult and necessitates the addition of salt or pH alteration to recover them after the water treatment process.
(2) Dispersion of nanocellulose in hydrophobic polymer matrices (membranes) remains a critical issue. However, the dispersion of nanocelluloses in polymer blends for sustainable wastewater treatment applications can be achieved by surface grafting of nanocelluloses with low molecular weight polymers. Solution-casting is the most important method for preparing nanocellulose-polymer composite membranes, which, however, remains difficult for large-scale application.
(3) Production of nanocellulose from plant sources is generally based on multi-step, top-down techniques that include physical (e.g., refining, mechanical grinding, ultrasonic grinding, thermal treatment), chemical (e.g., acid hydrolysis, alkali treatment, and chemical modification), biological (e.g., enzymatic hydrolysis and production of cellulose nanofibers from bacteria), and hybrid methods [43,44]. High water and energy consumption and yield are the main challenges in the preparation process, along with by-product toxicity [4]. For example, acid wastewater is typically generated from the washing process for neutralizing the pH value of the nanocellulose suspension [45].

**Table 2:** Relation between the physicochemical features of nanocelluloses and their adsorption capacity for hazardous pollutants in the aqueous environment.

Nanocellulose Features	Effect on Adsorption	Reference
High surface area	Increases the specific surface area from micro- to nano-size, thus enhancing the nanocellulose adsorption capacity. The mixed aerogel (ratio of 1:3 CNC/CNF) can provide a higher specific surface area than pure CNC or CNF.	[43,45]
High aspect ratio	Aspect ratio of CNC (10–80) is generally smaller than that of CNF (up to 80–500), depending on nanocellulose sources and the treatment process. Favors the set-up of percolated CNCs and entangled CNF networks held by strong hydrogen bonding, thus enhancing the adsorbent mechanical strength	[45]
High mechanical stiffness	CNC and CNF high mechanical stiffness (modulus), ~130 and ~70 GPa, respectively, increases the adsorbent material stiffness and cohesion. High crystalline forms (CNCs and CNFs) are transparent, and gas-impermeable with a very high tensile strength up to 8 times that of steel.	[6,46]
High crystalline degree	Nanocellulose high crystallinity degree (60–80%) enhances the adsorbent chemical resistance and reduces cellulose solubility even in high polar solvents	[47]
Susceptible to surface functionalization	Hydroxyl group functionalization (oxidation, esterification, etherification, radical grafting, and silylation) increases the nanocellulose adsorption capacity	[48,49]
Stability in water	Reduces biofouling of nanocellulose-based adsorbents. The surface of cellulose-based water treatment materials is negatively charged due to the high concentration of hydroxyl and carboxylate groups, resulting in higher electrostatic repulsive forces between the surface layer and most model foulant.	[50]
High surface tension	High surface tension (nanocellulose surface energy is ~60 mJ m^−2^) of nanocellulose-based adsorbents by water improve the wetting characteristics and reduce the bio-fouling	[51]

## 3. Adsorbents for Hazardous Metal Removal 

Hazardous metal ions (e.g., Ni^2+^, Ag^+^, Cd^2+^, Cu^2+^, Pd^2+^, Hg^2+^, U^6+^) originating from battery manufacturing, petroleum refining, metal plating drainage, mining activities, paint manufacturing, and photographic products, are abundantly released in the environment [51,52,53,54]. The pollution of agricultural soil causes the wide distribution of toxic heavy metals in the environment, and this affects the microorganisms and plants′ growth. Exposure to heavy metals (oral ingestion, inhalation, and dermal exposure into humans) can cause damage to the lungs, liver, kidneys, and other organs. Radioactive and heavy metal ions have been found to interact with cell components such as DNA and nuclear proteins, causing DNA damage. Prolonged exposure to toxic heavy metals causes cancers (i.e., prostate, stomach, kidney, urinary system, and bones) and Alzheimer’s disease [55]. From this perspective, it is necessary to develop green treatment strategies to remove hazardous heavy metals from the industrial water system [52].

**Figure 2:**
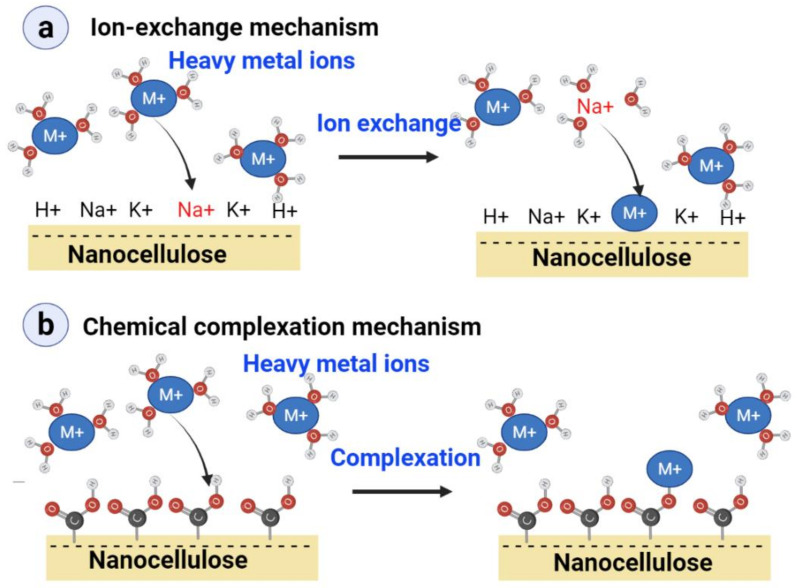
Heavy metal removal mechanism from water system using nanocelluloses: (**a**) Ion exchange mechanism which involves the adsorption of hazardous metal ions (M^n+^) takes the place of other ions (K^+^, Na^+^, H^+^) already associated with the nanocellulose surface; (**b**) chemical complexation mechanism in which the carboxyl (-COO^−^) and hydroxyl (-OH) groups of the nanocelluloses have specific site interactions with particular hazardous metal ions.

To date, several methods (chemical precipitation, adsorption, reverse osmosis, solvent extraction, and electrochemical treatment) have been employed to remove radioactive and heavy metals from contaminated water [53]. Adsorption of hazardous (radioactive and heavy) metal ions is considered as one of the suitable water treatment methods due to due to its high efficiency, low cost, and ease of operation. Numerous studies reported that the nanosorbents remove radioactive and heavy metals from wastewater, e.g., carbon tube, graphene oxide, polymeric, zeolites, metal and metal oxides nanosorbents [54]. For using nanocellulose-based adsorbents, ion exchange and chemical-complexation are the main two mechanisms concerned for the uptake of heavy metals (Figure 2). The ion-exchange mechanism involves the adsorption of hazardous metal ions (M^n+^) takes the place of other ions (K^+^, Na^+^, H^+^) already associated with the nanocellulose surface (Figure 2a). In chemical complexation, the carboxyl (-COO^−^) and hydroxyl (-OH) groups of the nanocelluloses have specific site interactions with particular hazardous metal ions (M^n+^) (Figure 2b). The maximum adsorption capacity of nanocelluloses is limited by their surface area, functionality, and stoichiometry rules which cannot exceed half the content of surface ionic sites. For this reason, increasing surface area and surface functionalization is necessary to increase or introduce more complexing sites on which the hazardous metal ions can be adsorbed. Most work related to the usefulness of nanocellulose as an adsorbent for hazardous metal ions involved CNF [55,56,57,58,59], while limited works have been reported on CNCs and BNCs. The high surface area and nature of the functional groups on nanocelluloses drive their sorption efficiency. Table 3 lists the various nanocelluloses used as adsorbents to eliminate hazardous metal ions from contaminated wastewater.

Anionic modification (carboxylic groups) believes to be the most prevalent method to boost the adsorption capacity towards hazardous metal ions removal. The higher numbers of negative binding sites the enhanced complexation and ion-exchange adsorption toward different hazardous metals ions. For example, the carboxylated CNFs can adsorb Pb^2+^, Cd^2+^, Ni^2+,^ and Cr^3+^ from aqueous solutions with an efficiency increase of 3–10% compared with unmodified CNFs [74]. Formation of anionic carboxylate groups with quite high densities (TEMPO-oxidized) on CNFs has been specifically used for the removal of radioactive uranyl ions (UO_2_^2+^). The adsorption values of TEMPO-oxidized CNFs are 2–3 times higher than those of traditional adsorbents (hydrogels and SiO_2_ NPs) [56]. Copper adsorption increased with pH and degree of TEPO-oxidation, with no noticeable adsorption at pH < 7. Since at low pH all carboxylic groups are protonated, and so cannot trap copper ions, as pH increases the volume of carboxylic acid entities decreases to the benefit of their negatively charged carboxylate counterparts, which can adsorb Cu^2+^ [58].

Sulfate functionalization (-O-SO_3_) of nanocelluloses (CNCs and CNFs) surface can be achieved by an acid hydrolysis reaction. Sulfuric acid is a cheap chemical to produce nanocellulose with sulfate groups. However, due to the highly concentrated sulfuric acid, the use of sulfuric acid leads to hydrolysis of the amorphous region and the formation of the CNCs rather than CNFs [57]. Using sulfonated CNF prepared from wheat straw pulp fine, Pb^2+^ was efficiently adsorbed from model solutions (1.2 mmol·g^−1^), whereas unmodified CNF demonstrated low adsorption capacities [54]. The unmodified CNCs had a greater ability to adsorb Ag^+^ (34 mg·g^−1^) than the CNFs (15 mg·g^−1^) due to the absence of surface sulfate groups [60]. In the remediation of cationic toxic metals from water bodies, CNCs were extracted from rice straw by using a mixture of HCl/H_2_SO_4_ acid [59]. The Cd^2+^, Pb^2+^, and Ni^2+^ ions were removed with adsorption capacity of 9.7, 9.42, and 8.55 mg·g^−1^, respectively, at a pH of 6 [61].

Functionalized bacterial nanocellulose (BNC) was investigated for wastewater treatment applications [84,87]. Based on the fact that, specific adsorption capacity (active adsorption sites) of untreated BNC is quite low due to the absence of surface charges [84,86]. The BNC fiber surfaces must be surface functionalized to enhance the specific adsorption and capture of hazardous metal ions and other pollutants. For instance, amino-functionalized BNC, which is synthesized through reaction with epichlorohydrin and diethylenetriamine, shows adsorption capacities of 63 for Cu^2+^ and 87 mg/g for Pb^2+^ at pH 4.5. However, the grafted amine group protonation at lower pH decreases significantly adsorption. After filtration, the adsorbed metals can be removed by recovery of the aminated BNC during the washes in ethylenediaminetetraacetic acid [87]. BNC chemically modified with ammonium sulfamate (NH_4_OSO_2_NH_2_) displays a maximum adsorption capacity for Pb^2+^ of 22.7 mg/g, which is five-fold higher than that of unmodified BNC [85]. Likewise, carboxymethylated BNC shows significantly higher adsorption values for Cu^2+^ and Pb^2+^ compared with unmodified BNC (12.6 mg/g for Cu^2+^ and 60.4 mg/g for Pb^2+^ versus 9.7 mg/g for Cu^2+^ and 22.6 mg/g for Pb^2+^ at pH = 4.5). These experiments also demonstrated that Cu^2+^ and Pb^2^ absorption is influenced by variations in pH, contact time, and concentration of the metal ions [84]. 

**Figure 3:**
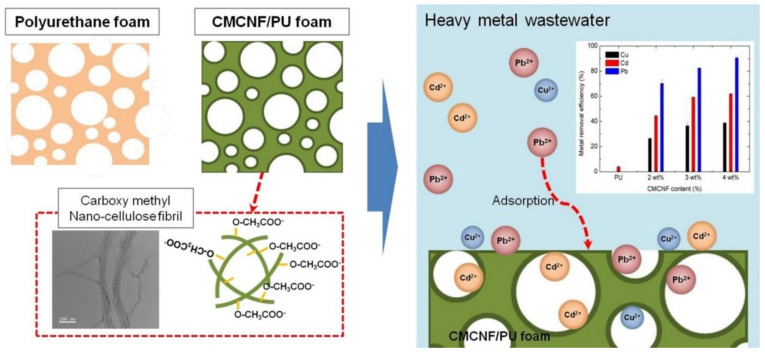
Fabrication of a template structure for carboxymethylated (CM) cellulose nanofibers (CNF) with polyurethane (PU) foam with controlled pore structure, for use as a modular adsorbent of heavy metals (Cd^2+^, Cu^2+^, Pd^2+^) in contaminated water [94], ©Elsevier, 2018.

Surface grafting of nanocelluloses (CNCs, CNFs, BNC) with functional organic molecules (3-aminopropyl triethoxysilane [77], oxolane-2,5-dione, succinic anhydride [88], ethylenediaminetetraacetic dianhydride [89]) have been recently used to enhance the removal of hazardous metal ions (Ni^2+^ Cu^2+^ and Cd^2+^) from contaminated water. Similarly, grafting of nanocelluloses with natural (chitosan [90], chitin [91], starch [92]) and synthetic (polyethyleneimine [93] and polymethacrylic acid-co-maleic acid [81]) polymers creates a higher number of active sites with a specific binding affinity for metal ions and consequently enhances adsorption yields [93]. CNF grafting with polymethacrylic acid-co-maleic acid^69^ and formation of aerogel via radical polymerization improves the adsorption exceeding 95% toward of Pb^2+^, Cd^2+^, Zn^2+^, and Ni^2+^ from wastewater, when their concentration was lower than 10 ppm. Surface grafting CNFs from bamboo with polyacrylic acid increases Cu^2+^ removal from aqueous solutions by 3-fold compared with unmodified CNFs [73]. Recent studies also evaluated the use of synthetic polymers (polyurethane foams, PU) as a template for the dispersion of carboxymethylated CNFs (Figure 3). The composite foams exhibited well dispersed carboxymethylated CNFs in PU matrices with highly porous structure, enhanced mechanical property, and excellent adsorption capacity for Cu^2+^, Cd^2+^ and Pd^2+^ ions removal as well as recyclability [94].

Combination of nanocellulose with other nanoadsorbents (e.g., carbon tube, graphene oxide, zeolites, metal, and metal oxides) have been examined for the capture and elimination of hazardous metal ions from contaminated water [95,96]. Carboxymethylated CNF/hydroxyapatite hybrids have been tested as adsorbents for hazardous metal ions (e.g., absorption capacity for Pb^2+^ ions of ~625 mg·g^−1^) [97]. Cellulose/montmorillonite composites showed high removal capacity (22.2 mg/g) for toxic bichromate anions (Cr^6+^). Similarly, electrospun cellulose acetate/organically modified montmorillonite showed high removal capacity (~20 mg·g^−1^) for Cr^6+^ removal [98]. Recently, graphene oxide (GO)/dialdehyde cellulose membranes cross-linked with triethylenetetramine [CH_2_NHCH_2_CH_2_NH_2_]_2_ showed maximum adsorption capacity values of 65.1 and 80.9 mg/g for Cu^2+^ and Pb^2+^ ions, respectively [99]. GO was also mixed with cellulose in ionic liquids and was tested for the specific adsorption of Ce^3+^. The hybrid cellulose/GO films, without an obvious interface between phases, display a maximum capacity of 109.1 mg/g for Ce^3+^ adsorption [100]. However, the GO incorporation with cellulose in ionic liquids for water purification applications has been hampered by difficulties due to the small size of GO particles that are difficult to recover [100,101]. 

Fabrication of magnetic nanocellulose based adsorbents with the aim of magnetic separation and reuse is a viable approach; it allows processing large effluent volumes and adsorbent regeneration [102]. Recently, hybrid Fe_3_O_4_/BNC nanocomposites have been used for the selective removal by magnetic separation of different hazardous metal ions in complex wastewater mixtures and show high adsorption capacity for Pb^2+^ (65 mg·g^−1^), Mn^2+^ (33 mg·g^−1^), and Cr^3+^ (25 mg·g^−1^) [75]. Similarly, aminated BNC/Fe_3_O_4_ NPs exhibit high adsorption rates for As^5+^ ions (90 mg·g^−1^) due to their high affinity for magnetic Fe_3_O_4_ NPs and amines [103]. A study on BNC composites in which magnetite Fe₃O₄ NPs were homogeneously distributed in the BNC matrix found that Cr^6+^ ion removal is strongly influenced by the medium pH, with the highest removal efficiency (5.13 mg·g^−1^) at pH 4 [104]. Spherical BNC/Fe_3_O_4_ particles, obtained by encapsulating magnetite Fe_3_O_4_ NPs of 15 nm in size into BNC particle, showed high adsorption capacities of 65, 33 and 25 mg/g for Pb^2+^, Mn^2+,^ and Cr^3+^, respectively [75]. Overall, these studies indicate that magnetic cellulose nanocomposites display excellent adsorption efficiency, compared with individual nanocelluloses. 

## 4. Adsorbents for Hazardous Organic Pollutants Removal 

**Figure 4:**
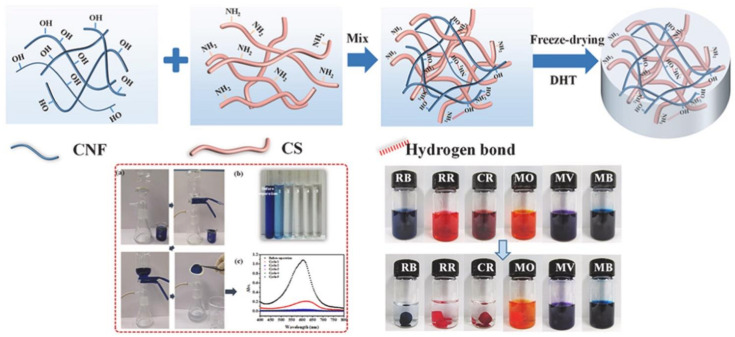
Highly efficient and selective removal of anionic dyes from water using a composite membrane of cellulose nanofibril (CNF)/chitosan (CS) prepared by de-hydrothermal treatment [125], © Elsevier, 2021.

**Figure 5:**
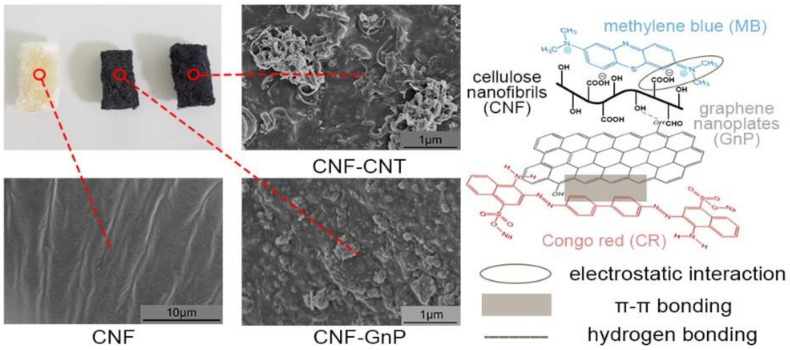
Cellulose nanofibers (CNF) and carbon nanotubes (CNT) or graphene nanoplate (GnP) hybrid aerogels for the adsorption and removal of cationic and anionic organic dyes [126], ©MDPI, 2020.

Hazardous organic pollutants (dyes, pharmaceutical compounds, pesticides, fertilizers, and petrochemicals) can pollute water bodies [105,106]. The application of nanocelluloses-based materials (adsorbent, photocatalysts, and filtration membrane) for treating wastewaters contaminated by hazardous organic pollutants has been largely discussed in the literature (Figure 4 and Figure 5), as summarized in Table 4. Generally, the affinity of native cellulose microfibers towards organic pollutants is 100 to 500 times lower than that of conventional nanomaterials, such as zeolite or activated carbon, due to the low number of active sites for interaction with the organic pollutants [107]. Alternatively, surface-modified nanocelluloses have been tested as support materials for the adsorption of various organic pollutants [39,107,108,109,110,111,112,113,114,115,116,117,118,119,120,121,122,123,124]. This is mainly explained by their robust mechanical properties, the high specific surface area that allows creating active interaction sites after functionalization, and the small pore size of their filters/membranes. As the nanocellulose intrinsic hydrophilicity is not suitable for the adsorption of organic molecules, surface modifications, and/or formation of nanocomposite materials (e.g., porous films or aerogels with controllable porosity) are required to improve the adsorption and filtration capacity. 

Organic pollutants such as dyes, drugs, and pesticides are toxic even at small concentrations (l mg L^−1^) present in the environment. Organic pollutants in polluted water can cause several health problems to humans including skin rashes, headaches, diarrhoea, muscle and joint pain, difficulty breathing, irregular heartbeat [140,141]. Oral ingestion and inhalation of organic pollutants come under acute toxicity and may lead to different types of cancers, skin irritation, and allergic reactions [142]. Recently, nanocelluloses have been also investigated used as a potential adsorbent to eliminate pesticides, fertilizers, and drugs as contaminants. Bouli et al. (2018) reported that carboxymethyl nanocellulose hydrogels, filled with nanoclays are beneficial adsorbents, soil conditioning agents, and nutrient carriers, especially to improve the growth of cucumber [109]. Likewise, Mautner et al. (2017) demonstrated that the cationic cellulose nanopaper membranes are efficiently useful for the continuous removal of nitrates, released from the chemical fertilizer in the agriculture soil and irrigation water [108]. Similarly, several researches revealed that the nanocellulose can serve as a potential adsorbent for the removal of pesticides, such as chlorpyrifos [143], tetraconazole [144], diuron [145], organophosphorus [146], difenoconazole, and nitenpyram [147]. Nanocelluloses are also employed for the adsorption and elimination of hazardous drugs from industrial wastewater, such as ciprofloxacin, diclofenac [124], metronidazole [148], and ceftriaxone [149]. Nanocelluloses on their own and after anionic functionalization, can be used for cationic organic pollutants adsorption from wastewater. CNFs prepared from kenaf fibers were tested for the adsorption of cationic dyes. The very high adsorption capacity (123 mg/g at pH 9) can be explained by the specific CNF morphology that depends on the selected source [138].

Dyes are particularly dangerous, primarily due to the fast growth of textile manufacturing that consumes about 7 × 10^5^ tons of dyes per year [129]. Dyes are generally non-biodegradable, leading to a severe problem for plants and foods. Direct dyes are water-soluble anionic dyes, have a strong affinity with nanocelluloses. Acid dyes are water-soluble anionic dyes with high very poor affinity to bind nanocelluloses. The attraction forces between the chromophores of direct and acid dyes with nanocelluloses include hydrogen bonding, dipolar forces, and non-specific hydrophobic interactions. Typically, cationic modification of nanocelluloses increases the uptake of the direct and acid dyes. In contrast direct and acid dyes, sulfur dyes (–S–, –S–S–, –Sn–) and azoic (−N=N−), vat dyes are water-insoluble and they are mainly having a high affinity to be adsorbed with nanocelluloses. 

Surface modification of nanocelluloses develops functional nanocellulose-based materials and enhances the organic adsorptive property [124,150,151,152]. To make nanocellulose adsorbents for anionic dye, chemical modifications, such as the introduction of positively charged (cationic modification with amino groups), are usually required (Figure 4). Anionic dyes can be separated using cationic CNFs, obtained by surface quaternization with glycidyl trimethyl ammonium chloride [139]. Several studies focused on the selective adsorption onto cationic CNFs of negatively charged water organic pollutants (e.g., phosphates, nitrates, fluorides, and sulfates) [153]. For instance, CNFs have been grafted with soy proteins to enhance their bioactivity towards biomimetic mineralization. After precipitation of rod-like hydroxyapatite particles, the composite material exhibited high adsorption of methylene blue (up to 454 mg/g), cost-effectivity, and reusability. This suggests that the soy protein-grafted CNF/hydroxyapatite nanocomposite is a promising adsorbent for water purification [119]. A multifunctional Fe-aminoclay/CMC/polyhedral oligomeric silsesquioxane composite was developed to eliminate cationic dyes from contaminated sites. The composite exhibits adsorption of 438 mg/g for methylene blue and 791 mg/g for Chrysoidine G. Its good adsorption capacities are explained by its layered structure and by the presence of amino groups (on the clay surface) and carboxylate/hydroxyl groups (on the CMC backbone) that contribute to the dye adsorption through electrostatic attraction and ion exchange [154]. 

Combination of the nanocelluloses with inorganic nanoadsorbents (nanoclay, zeolite, nano-hydroxyapatite) has the advantage of eliminating organic pollutants because of the availability of materials as adsorbents [97,121,155]. For instance, Nanohybrids of carboxymethylcellulose (CMC)/hydroxyapatite (HAp) NPs were formed via an in-situ synthetic approach and displayed an adsorption capacity of 200 mg·g^−1^ for acid yellow 220 removals from aqueous solutions [121]. In another study to increase the functionality of polysaccharides, carboxymethyl cellulose (CMC) grafted with polymethacrylic acid (MAA) was used as a growth modifier to enhance hydroxyapatite mineralization. The CMC/MAA/HAp hybrid material efficiently removes methylene blue with an adsorption value of 671 mg/g [97].

Magnetic nanoparticles can be deposited onto nanocelluloses (CNFs, CNCs, and BC) via in situ hydrolyses of metal precursors that have been investigated for dye removal [156]. The effects of different concentrations of nanocelluloses on the morphology and the magnetic properties of the nanocellulose based materials produced have been discussed in the literature [120,156,157]. For example, spherical magnetic CMC/Fe_3_O_4_ NPs (NP diameter of 25 nm and CMC from mesquite tree pulp) were investigated as biodegradable adsorbents for organic dyes (methylene blue) in wastewater [120]. Likewise, magnetic nanofibers, prepared by electrospinning cellulose acetate nanofibers and co-precipitation of immobilized ZnO and Fe_3_O_4_ NPs, show an enhanced capacity to absorb phenol from aqueous solutions (64% in 2 h) [157]. Complex nanocomposites of cyclodextrin-modified CNC in combination with paramagnetic Fe_3_O_4_/SiO_2_ nanorods served as an efficient separation tool for procaine hydrochloride (anesthetic drug) and imipramine hydrochloride (antidepressants drug) [158]. The latter field is believed to become an extremely potential domain that is currently under fast development in view of public health issues.

Highly porous nanocellulose gels (hydrogels, aerogels, organo-gels, sponges-like materials) were prepared from nanocelluloses (CNCs, CNFs, BNC) and their adsorption properties towards a wide range of organic pollutants were investigated [159]. A novel, very robust porous 3D sponge was prepared from reduced graphene oxide (rGO), vitamin C, and CNCs. Vitamin C was used as a reducing agent and as a soft template that allowed creating a unique hierarchical structure with a multitude of interconnected and homogeneously distributed pores, even in the sponge core. This specific structure resulted in a very high surface area for the uptake of methylene blue in water (850 mg/g) [160]. This is much better than what obtained using cellulose/rGO fibers, prepared with the wet-spinning technique (480.8 mg/g) [161]. Hybrid cellulose aerogels assembled from CNFs with carbon nanotubes and graphene nanosheets have been used to remove cationic (methylene blue) and/or anionic (Congo red) dyes [126]. Comparison of the hybrid aerogel structural properties and adsorption mechanics in the function of the CNF/GnP ratio indicated that the optimum performance is obtained with a CNF/graphene nanosheets ratio of 3:1. For the single dye system, the adsorption kinetics for methylene blue (maximum adsorption: 1178 mg·g^−1^) and Congo red (maximum adsorption: 585 mg·g^−1^) are explained by pseudo-second-order adsorption kinetic and the monolayer Langmuir adsorption isotherm. For the binary dye system, the CNF/graphene nanosheets hybrid displays better dye adsorption capacity compared with pristine CNFs and graphene nanosheets. Moreover, almost 80% of methylene blue or Congo red can be desorbed from CNF/GnP using ethanol as a desorption agent. In conclusion, aerogels from nanohybrid materials made of CNF with carbon nanotubes or GnP are promising reusable adsorption materials for dye removal (Figure 5). Table 4 gives an overview of the different nanocellulose types and surface modification procedures, highlighting the variety of adsorption material for organic dyes [126].

## 5. Wettable Materials for Oil/Water Separation 

**Figure 6:**
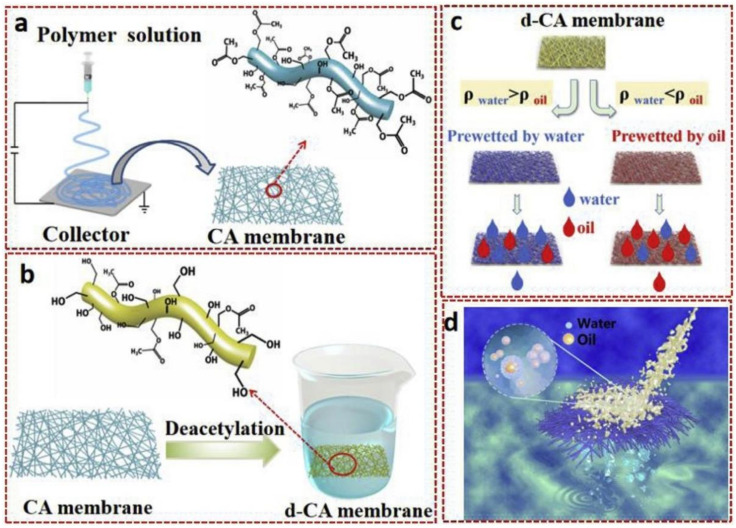
Formation of dual super-amphiphilic modified cellulose acetate nanofiber membranes by electrospinning, with highly efficient oil/water separation and excellent antifouling properties, (**a**) Electrospinning process to prepare cellulose acetate nanofiber membrane; (**b**) Deacetylation process to form d-CA; (**c**) Prewetted process to form amphiphilic structure; and (**d**) super-amphiphilic structure to separate water and oil [163]. ©Elsevier, 2020. CA, cellulose acetate nanofiber; d-CA, dual super-amphiphilic modified cellulose acetate nanofiber.

Oil-based compounds from chronic discharges and oil spills represent an important source of environmental pollution. Oil toxicity is mostly related to the aromatic fraction made of both low molecular weight (LMW)/high molecular weight (HMW) polycyclic aromatic hydrocarbons (PAHs) ratio [162]. As well, dissolved fractions of petroleum appear to be more bioavailable to aquatic organisms. Techniques to separate oil/water mixtures are crucial for many industries (e.g., food, pharmaceutical, and petrochemical) that use and produce huge amounts of oils and require solutions for their removal from wastewater [163]. Normally, efficient water/oil separation is accomplished by separating water and oil, followed or in combination with oil adsorption. Currently, the most common water/oil separation methods (sedimentation, centrifugation, or hydrocyclone) are based on differences in gravity or density [163,164]. Recently, nanocelluloses (CNFs and CNCs) incorporation in membranes for oil separation offers interesting opportunities, together with the (partial) modification of the original hydrophilic surface properties towards higher hydrophobicity and/or balanced amphiphilicity [165]. Besides CNFs and CNCs, electrospun cellulose nanoyarn membranes with grafted hydrophobic moieties have been used for oil removal. The step-by-step fabrication of oil/water separation membranes by electrospinning of cellulose acetate, followed by their deacetylation, is shown in Figure 6 [163]. Interestingly, electrospun cellulose acetate nanofibers are super-amphiphilic in air, oleophobic in water, and super-hydrophilic in oil. The membranes are characterized by high separation flux (up to 38,000 L/m^2^·h) and gravity-based separation efficiency (up to 99.97%) for chloroform/water mixtures. Moreover, their anti-pollution and self-cleaning capacities boost their recyclability. Therefore, cellulose acetate nanofiber membranes could be interesting for chemical plants, textile mills, food industry, and also offshore oil spills [163].

**Figure 7:**
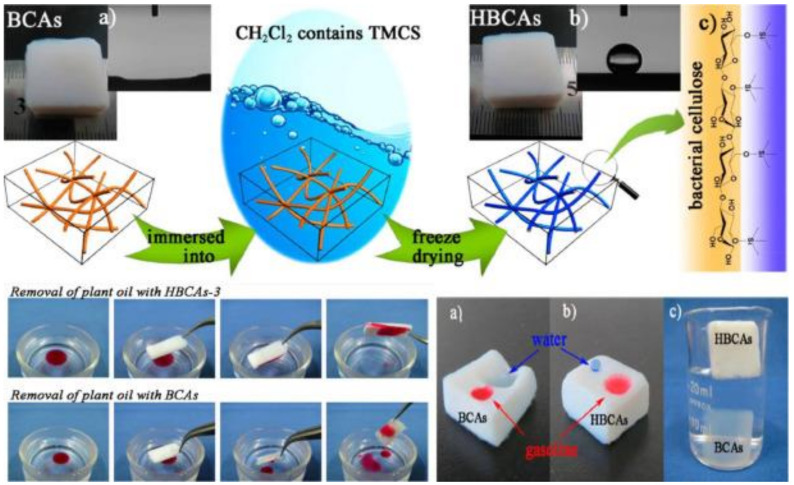
Surface hydrophobization of bacterial cellulose acetate membranes with trimethylchlorosilane for the efficient removal of plant oil from water [169], ©American Chemical Society (2015). BCA—bacterial cellulose acetate; TMCS—trimethylchlorosilane; HBCA—hydrophobic BCA.

Highly porous nanocellulose gels and sponge-like materials prepared from nanocelluloses (CNCs, CNFs, BNC) are frequently exploited for oil/water separation due to their high oil adsorption capacity thanks to their porous structure [165]. For example, CNFs can be modified with silanes to make them hydrophobic and to adsorb oil contaminants from the water surface [166]. Likewise, CNF aerogels coated with a thin layer of TiO_2_ NPs by sol-gel chemistry are efficient for oil adsorption [167]. Moreover, silylated nanocellulose sponges provide a flexible, hydrophobic, and ultra-light structure for oil removal from water mixtures [164]. These highly porous (up to 99%) silylated CNF sponges were obtained, using a freeze-drying method, from the pulp powder of oat straw cellulose through reaction with methyltrimethoxysilane. These sponges are more flexible than common inorganic porous materials, as indicated by the 96% of maximum shape recovery after compressive deformation. Their oleophilic and hydrophobic features allow the efficient and selective removal by adsorption of dodecane spills from surface water [164]. Similarly, ultralight carbon aerogels prepared from hydrophobized cellulose microfibers can be valuable for selective oil sorption [168]. These sponges show 99% of porosity, 0.01 g/cm^3^ density, good hydrophobicity, reusability, and very high adsorption capacity (>86 g/g) towards paraffin oil. Surface hydrophobization of BNC membranes has been modified with trimethylchlorosilane for the efficient removal of plant oil from water [169], by dipping BNC aerogels into liquid phase trimethylchlorosilane followed by freeze-drying (Figure 7). The obtained very hydrophobic (water/air contact angle as high as 146.5°) and highly porous (≈ 99.6%) surface-modified BNCs offer high selectivity for oil adsorption from water, with absorption capacities up to 185 g/g [169].

Incorporation of magnetic nanoparticles onto nanocelluloses via blending or in situ hydrolysis of metal precursors that have been used for oil removal from wastewater. Magnetic nanocellulose aerobeads, fabricated by freeze-drying of iron oxide (Fe_3_O_4_)-containing spherical CNFs (from used cardboard boxes), also show very good selectivity towards oil removal. These aerobeads (0.005 g/cm^3^ of density and 99% of porosity) possess excellent absorption efficiency towards various oils and organic solvents, especially castor oil (279 g/g). They also show superior recyclability and can be reused at least 10 times with high absorption capacity towards diesel oil (101 g/g) [170]. Fe_3_O_4_-based nanocellulose aerogels fabricated using the freeze-drying method can be used for the absorption of spilled oil in water. These aerogels have a density of 9.2 mg/cm^3^ and 68 g/g of adsorption capacity towards cyclohexane, with good absorption also of ethyl acetate, and vacuum pump oil [171]. This study indicates that magnetic nanocellulose aerogels and their nanocomposites can efficiently adsorb and eliminate oil from contaminated water; however, their biocompatibility and environmental degradability must be thoroughly investigated before their utilization for large-scale and long-term applications.

## 6. Flocculants and Coagulants for Suspended Materials 

**Figure 8:**
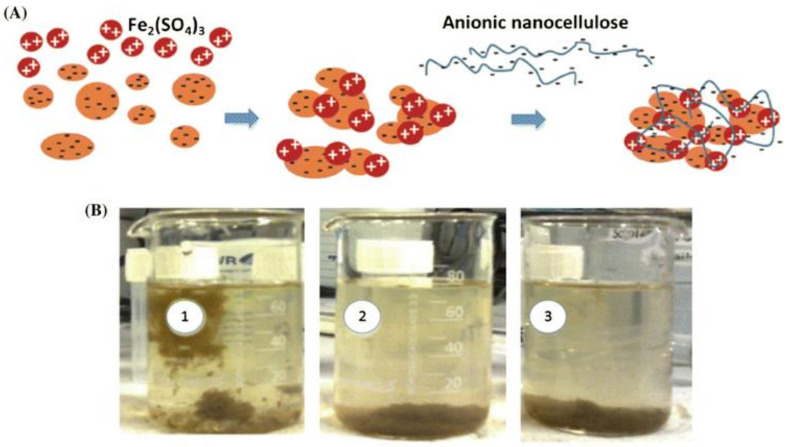
Flocculation mechanism of anionic nanocellulose to remove pollutants from water. (**A**) Binding and flocculation of cationic pollutants, and (**B**) visual observation of flocculation efficiency [10], ©Springer, 2017.

Flocculants are agents that can promote flocculation of colloids and suspended particles in liquids to aggregate them for floc formation [172,173]. Nanocelluloses such as CNF and CNC their modified counterparts have been investigated as flocculants for the treatment and elimination of contaminants from wastewater. These nanoflocculants induce flocculation of the suspended particles in contaminated wastewater by neutralizing the surface charge of particles or by forming bridges between the individual suspended particles. Figure 8 explains how anionic nanocelluloses (CNFs and CNCs) function as flocculants for the capture and removal of charged pollutants [10]. In contrast to native cellulose, there are several characteristic features of nanocelluloses (CNFs and CNCs) make them an ideal flocculants candidate for water treatment: (1) small size and high-surface-area rod-like morphology that give rise to percolation at low concentrations; (2) CNFs and CNCs can improve the formation of flocs compared to native fibers. In comparison between CNFs and CNCs, the higher electrostatic repulsion and rigidity of CNCs than CNFs will prevent the occurrence of physical and chemical entanglements contributing to reduced risk of gelation.

To date, there are only very few studies describing the applications of nanocelluloses (CNCs and CNFs) as flocculants in wastewater treatment; some examples are given in Table 5. Suopajarvi et al. (2013) fabricated carboxylated CNFs as anionic flocculants for municipal wastewater treatment. The high and long-lasting stability of anionic CNFs in aqueous suspensions provided excellent performance (turbidity reduction of 40–80% and COD removal of 40–60%) within the desirable pH range from 6 to 8 [174]. Likewise, Korhonen and Laine (2014) examined CNF/polyelectrolyte with different charge density for retention and flocculation of kaolin and calcium carbonate fillers in the papermaking industry. They showed that the flocculation efficiency is increased from 80% to 95% in the case of CNF/polyelectrolyte. The presence of polyelectrolytes induces the formation of CNF/polyelectrolyte bridges between kaolin and calcium carbonate particles and leading to efficient flocculation [175]. A recent study assessed the flocculation performance of hyperbranched cellulose grafted with polyethyleneimine (C_2_H_5_N)_n_ for the treatment of kaolin-contaminated wastewater. This cellulose-based flocculent decreased the residual turbidity of kaolin suspension from original 490 NTU to 4 NTU under 2.4 mg/L of the flocculent at pH 7.0 for 30 min [176]. 

Recently, Kemppainen et al. (2016) produced sulfonic acid and dicarboxylic acid cellulose (anionic) CNCs for the flocculation of quartz and hematite suspensions in contaminated water. The most effective performance was obtained at a pH of 8–9, and a carboxylic modified CNFs at dosage of 200–500 ppm was enough to flocculate hematite efficiently. The sulfonated modified CNFs is efficient a hematite flocculant as carboxylic modified CNFs at a dosage of 500 ppm after longer conditioning time and less vigorous stirring [177]. Campano et al. (2019) fabricated cationic CNCs as a novel flocculant for kaolinite/clay suspensions. The fastest flocculation values (10–30 mg/g) and biggest floc size were near the isoelectric point [178]. Yu et al. (2016) used microcrystalline cellulose (MCC) to fabricate carboxylated CNCs (length of 200–250 nm and diameter of 15–20 nm) by citric-hydrochloric acid hydrolysis. They could use these CNCs as a flocculant to remove cationic dyes and kaolin from suspensions with 99.5% of turbidity removal capacity [128].

**Table 5:** Various nanocellulsoes-based flocculants used for the water treatment process.

No.	Nanocellulsoe Flocculants	Contaminants	Optimum Flocculation Conditions	Highest Removal Efficiency	Ref. No.
1	Pristine CNCs	*Pseudomonas aeruginosa* (Gram-negative Bacteria)	Flocculant concentration (CNC:Bacteria) = 100,000:1, reaction time = 24 h	100%	[179]
2	Carboxylated CNCs	Kaolin clay (suspended filler particles)	Flocculant concentration = 40 mg L^−1^, pH = 4–10, reaction time = 30 min	95.4%	[128]
3	Pyridinium grafted CNCs	*Chlorella vulgaris* (Microalgae)	Flocculant concentration = 0.1 g flocculant per g microalgae, pH = 4–11, reaction time = 30 min	95%	[180]
4	Amine-functionalized CNCs	Sodium dodecyl sulfate (anionic surfactant)	Optimum pH = 4,	-	[181]
5	Carboxylated CNFs	Suspended particles	Flocculant concentration = 2.5–5.0 mg.dm^−3^, pH = 6–8, reaction time = 30 min	40–80%	[174]
6	Sulfonated CNFs	Suspended particles	Flocculant concentration = 2.5 mg dm^−3^, reaction time = 30 min	-	[182]
7	Quaternized CNFs	Reactive orange 16 (reactive dye)	Flocculant concentration = 150 mg.L^−1^, reaction time = 12 h	q_max_ = 0.477 mmol.g^−1^ for the reactive dye	[183]

## 7. Photocatalytic Materials for Hazardous Pollutants Degradation 

**Figure 9:**
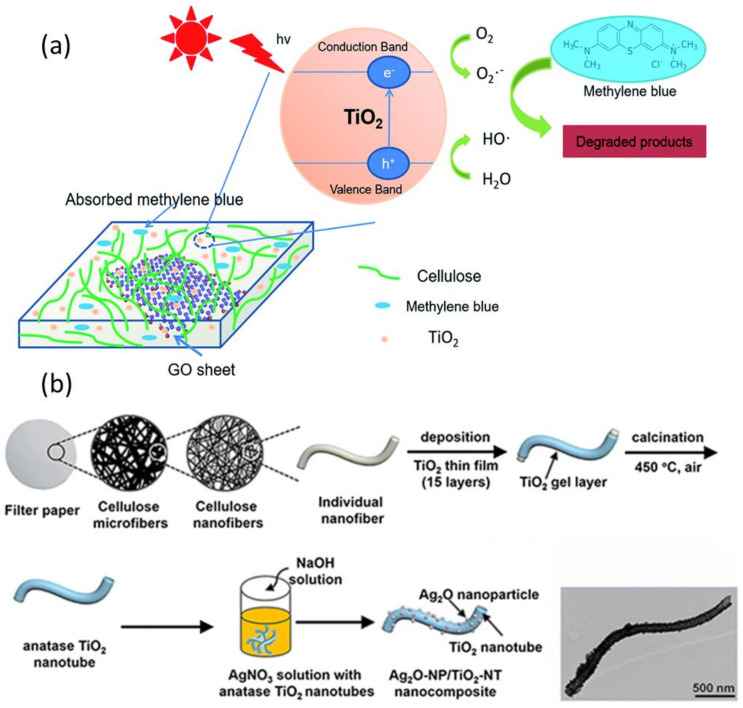
(**a**) Mechanism for photocatalytic treatment and degradation of an organic pollutant (methylene blue) in wastewater using graphene oxide (GO)/cellulose/TiO_2_ nanocomposites [190], ©Royal Society of Chemistry, 2020. (**b**) Deposition of TiO_2_ nanotubes and AgO_x_ nanoparticles (NPs) onto cellulose nanofibers (CNFs) for improved photocatalytic cleaning of wastewater [191], ©Springer, 2019.

Photocatalysis has emerged as an environmentally friendly, low-cost, and handy approach for the degradation of organic pollutants and/or dyes in wastewater. Even though nanocelluloses alone exhibits limited photocatalytic activity under the visible light-UV region, conventional metal oxide photocatalyst (ZnO [184] and TiO_2_ [185]) have been added to enhance the photocatalytic activity. These photocatalytic materials (individual particles, thin-film, membranes) have been developed using cellulose-based metal oxide nanostructures in the form of under UV and visible light irradiation. Photocatalysis harnesses photon energy to produce active radicals that promote the decomposition of organic pollutants [186]. When the photon energy is above the semiconductor energy gap, semiconductor materials can generate active hydroxyl radicals, leading to the excitation of electrons from the valence band into the conduction region [187]. This creates a hole that can generate hydroxyl radicals in an alkaline medium [188]. These radicals are highly reactive and can enhance the oxidation of recalcitrant chemicals in wastewater, leading to complete degradation of organic pollutants into nontoxic byproducts (i.e., CO_2_ and H_2_O) [189]. Active hydroxyl free radical radicals attack organic materials via four known pathways: radical addition, hydrogen abstraction, electron transfer, and radical combination (Figure 9a) [190]. The main challenge associated with semiconductor NP use in photocatalysis is their removal from the liquid after the reaction. Poor visible light absorption nanocellulose/TiO_2_ and nanocellulose/ZnO is the limitation of such photocatalysts due to the wide bandgap. Similarly, separation of these nanocomposites is difficult from reaction mixture due to non-magnetic characteristics.

Narrow bandgap (2–2.4 eV) semiconductors such as CuO_2_, CdS, Fe_2_O_3_ are recommended to generate more electron−hole pairs under visible light [192]. Incorporating co-catalyst NPs (Ag, AgOx, graphene) can narrow the bandgap and further improve the photocatalytic performance of wide bandgap photocatalysts in the visible region (Figure 9) [193,194]. Ferromagnetic photocatalysts such as Fe_2_O_3_ NPs can give the required optical absorption at the visible region with relatively narrow band gaps (~2.1 eV). More interestingly, their nanocomposite with nanocelluloses can be easily recovered from wastewater for recycling and further use, thanks to their unique magnetic properties [195]. Jiao et al. (2018) showed that a magnetic Fe_3_O_4_/CNF composite aerogel is an effective catalyst for the Fenton-like degradation of Rhodamine B (100% of removal efficiency) [196]. This catalyst was re-used for six successive runs and retained 97% of its removal capacity [197]. Novel cellulose-based photocatalysts have been developed with appropriate band gap (2.4 eV) and excellent photocatalytic activity and stability under UV-Vis light [198]. BiOBr exhibit narrow bandgap of 2.4 eV and high phenol degradation rate under visible light when using BiOBr/regenerated cellulose than BiOBr particles (80% versus 45% after 3 h of irradiation) [198].

Porous nanocellulose materials (cellulose aerogels and cellulose sponge) loaded with photocatalyst NPs have been also transformed into photocatalytic carbonaceous materials by hydrothermal treatment followed by thermal pyrolysis, among others [199]. The photocatalytic properties of such photocatalytic carbonaceous materials are enhanced because of their high surface area and porosity as well as their high ability for adsorbing organic pollutants [200]. Photocatalytic cellulose-based nanopapers have been also investigated as a novel support material for photocatalyst NPs immobilization. Matsubara et al. (1995) prepared TiO_2_-containing papers for the catalytic degradation of acetaldehyde under ultraviolet light [178]. Electrospun cellulose acetate membrane loaded with TiO_2_ (5 wt.%) show very high dye removal by photocatalytic degradation [185]. The resulting TiO_2_/cellulose composites efficiently degrade phenol (90% after 4 h) under weak ultraviolet light irradiation [195]. Table 6 lists the various nanocellulose/inorganic composites that have been used for wastewater treatment by adsorption or photocatalytic degradation.

## 8. Membrane Materials for Wastewater Treatment 

Membrane technology has emerged as a favorite processing and separation method (filtration, extraction, and distillation) for reclaiming water from salty and contaminated water streams for their re-use by humans. The use of nanocellulose based membranes for water purification and/or desalination is a highly efficient and environmentally responsible method because of the low energy consumption and high selectivity. The operational characteristics of nanocellulose based membranes are mostly determined by two parameters: their water permeation flux value, and their specific pollutant removal capacity. The nanocellulose pore size and surface conditions are important parameters for controlling the filter efficiency. The membrane rejection might be explained by two mechanisms: (i) size-exclusion effects, and (ii) the membrane chemical or physical affinity for the pollutant. In the first mechanism (size-exclusion), the pollutant is selectively rejected depending on its size relative to the membrane pore size. The functioning and efficiency of nanocellulose based membranes depend on their selective retention of pollutants. Depending on the pollutant size, cellulose-based membranes are categorized into [195]:

(1) Microfiltration membranes with microspores of 10 µm to 100 nm; to be used for removal of suspended particles and Bacteria (200 nm–30 micron)
(2) Ultrafiltration membranes with nanopore range from 100 nm to 2 nm; it is suitable for removal of nanoparticles and viruses with sizes 50–200 nm
(3) Nanofiltration membranes with nanopore size range from 2 nm to 1 nm; to be used for removal of organic pollutants (0.7–1.5 nm)
(4) Reverse osmosis with sub-nanopore size from 0.1 to 1 nm; to be used for hazardous metal ion (0.1–0.7) removal.

Nanocellulose membranes predominantly operate according to the size-exclusion mechanism, and the pore size depends on the formation of a random CNF or CNC network that generates the “micro/nano-pores”. The pore size (micro/nanosize) can be modulated through variations in the aspect ratio and choice of nanocellulose material (CNFs/CNCs) and processing steps (thickness and dense packing). However, it is worth noting that small pore sizes generate higher pressures during the transportation of the effluent flow through the membrane [11]. Based on a theoretical pore flow model, pollutants with sizes larger than the mesh of the nanocellulose based membranes are captured by the size exclusion mechanism. Conversely, the rejection mechanism for smaller pollutants is mainly governed by the pollutant solution-diffusion mode. This does not often occur with nanocellulose based membranes. The second removal mechanism (affinity) depends on the electrostatic interactions between the pollutant and the surface functionalities of the nanocellulose based membranes. The surface modifications have been successfully used to tune nanocellulose reactivity towards different water pollutant types (e.g., biological species, inorganic pollutants, heavy metals). The modification processes include and are not limited to: (1) carboxylic acid halides create ester linkages, (2) sulfuric acid treatment provides sulfate esters, (3) epoxides create ether linkages, (4) acid anhydrides create ester linkages, (5) TEMPO mediated hypochlorite oxidation creates carboxylic acids, (6) isocyanates create urethane linkages, (7) chlorosilanes create an oligomeric silylated layer; (8) halogenated acetic acids create carboxymethyl surfaces [216]. The chemical surface modifications modulate the nanocellulose hydrophobic and hydrophilic properties because its intrinsic hydrophilicity contributes to the swelling and loss of mechanical strength or stiffness of the filter material. High hydrophobicity might increase the flow resistance and/or modify the selectivity for the adsorbed species.

**Figure 10:**
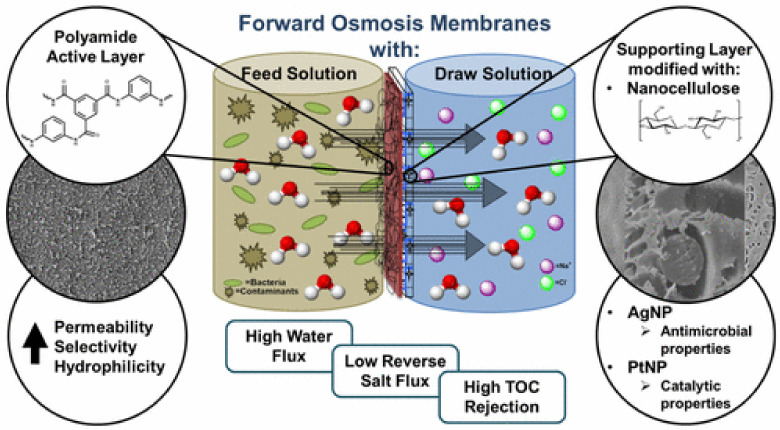
Enhanced performance of nanocellulose filter membranes for forwarding osmosis through modification of the nanocellulose material surface by coating with a polymer (e.g., polyamide active layer), and by nanoparticle (NP) deposition (e.g., Ag, Pt) [220], ©American Chemical Society, 2017. TOC, total organic carbon.

To date, three distinct techniques have been investigated for the fabrication of nanocellulose membranes: (i) electrospinning of cellulose mats and impregnation [217], (ii) vacuum filtration of the nanocellulose suspension and coating [218], and (iii) freeze-drying of the CNF suspension in the presence of an additional matrix component [131]. In parallel with papermaking, vacuum filtration is the most straightforward and feasible method for scaling up and for the fabrication of nanopaper membrane that can then be deposited onto a carrier substrate or used as free-standing units [218]. The final structure can be stabilized, in combination with hot-pressing to add a matrix material, to fabricate fully bio-based membranes. The obtained nanopapers can be used as membranes to efficiently adsorb organic compounds, phosphates, nitrates, fluorides, metal ions, or biological substances (e.g., bovine serum albumin) [219]. The efficiency of nanocellulose membrane can be enhanced by adding specific chemical modifications (e.g., coating, grafting), and/or by deposition of functional NPs. Figure 10 shows enhanced performance of nanocellulose membranes for forwarding osmosis through modification of the nanocellulose membrane by coating with a polymer (e.g., polyamide active layer), and by nanoparticle (NP) deposition (e.g., Ag, Pt) [220].

**Figure 11:**
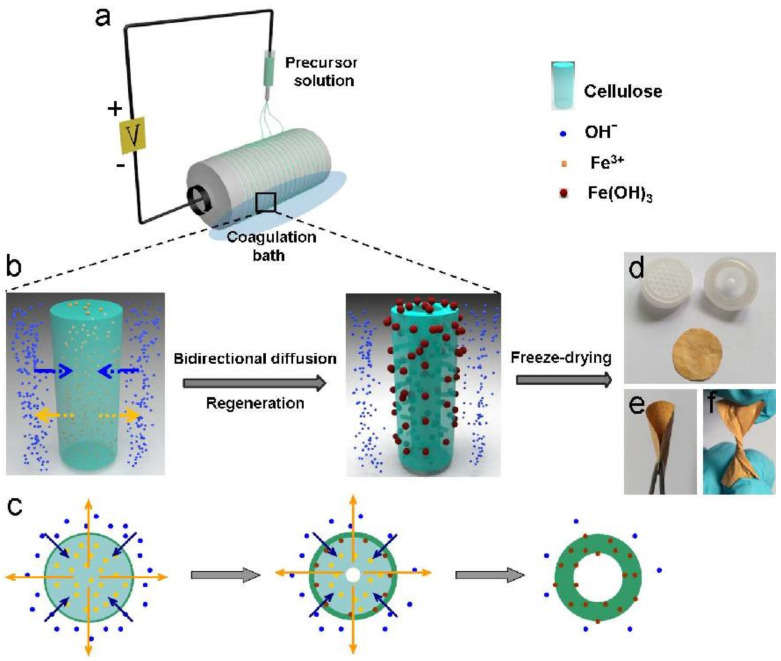
Electrospinning with suitable solvent systems was used to fabricate hybrid nanocellulose fibers with solid or hollow core and deposition of Fe(OH)_3_/cellulose nanoparticles: (**a**) Schematic representation of the electrospinning process for nanofiber fabrication; (**b**,**c**) Schematic representation of the hollow fibers as membranes for diffusion and regeneration, respectively; (**d**–**f**) Photographs of the Fe(OH)_3_@Cellulose HNFs membranes and the dead-end filtration device, [223] ©American Chemical Society, 2017.

Electrospun cellulose nanofiber membranes (ENMs) are a cutting-edge membrane technology that offers a high rejection rate compared to conventional membranes. As shown in Figure 11, hollow Fe(OH)_3_/cellulose can be obtained by electrospinning using a suitable solvent system, whereby the solvent exchange mechanism is induced and solid or free core structures are created [221]. Concomitantly, Fe(OH)_3_ NPs are formed and deposited onto the cellulose membrane structure during the in situ reductions of FeCl_3_ added to the spinning solution. The fiber size and diameter can be controlled by modifying the spinning parameters (e.g., applied voltage and concentration ratios). Moreover, increasing NaOH concentration in the coagulation bath leads to larger fiber diameters and surface pore sizes. The hybrid nanocellulose fibers fabricated with this method display extremely specific surface area and many active species for the interaction with effluents, and consequently good removal activity of organic dyes [221]. Similarly, Yang et al. (2014) developed composite membranes from CNFs functionalized with thiols that were dimensionally stabilized and fixed after cross-linking into an electrospun polyacrylonitrile nanofibrous scaffold during thermal curing. They tested these membranes in an ultrafiltration set-up for Cr^6+^ and Pb^2+^ removal with satisfactory results [222]. Similarly, Wang et al. (2013) prepared nanofibrous membranes containing CNCs by electrospinning to remove *Brevundimonas diminuta* and *Escherichia coli* bacteria from water in conditions of relatively high flux rate and low drop in pressure [223]. 

**Figure 12:**
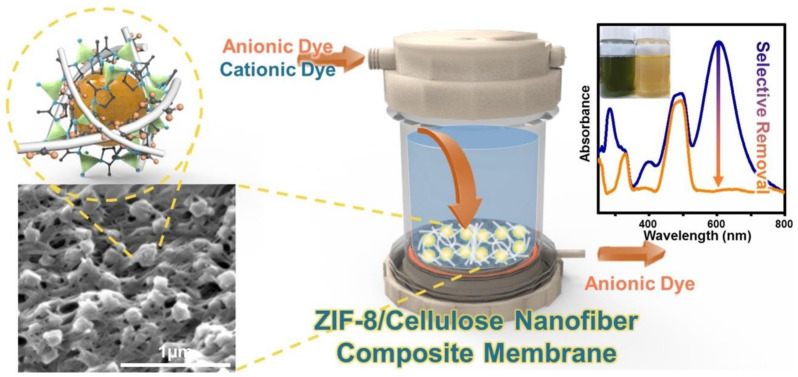
Control of the cellulose nanofibrous composite membrane structure by in-situ synthesis of zeolitic imidazolate framework-8 (ZIF-8) at cellulose nanofibers (CNF) for the selective removal of cationic dyes [235], © Elsevier, 2019.

Limiting fouling by the addition of biological and/or organic moieties is a crucial issue to ensure the nanocellulose membrane’s durability. CNFs and CNCs hydrophilicity and easily functionalizable surface are important features to retard and/or prevent fouling [224]. Membranes prepared by CNF incorporation into composites with a polymer matrix, such as cellulose acetate [225,226], polyethylene oxide [227], polyvinyl alcohol [228,229] polypyrrole [230], poly (ether sulfone), and polyhydroxybutyrate [231], have been tested for the removal of heavy metals. Moreover, composite membranes obtained by incorporating zeolitic imidazolate framework-8 (ZIF-8) in CNFs show high durability for the selective removal of 99% of cationic dyes from anionic and cationic dye mixtures (Figure 12). Likewise, CNF filter papers from microfibrillar *Cladophora* cellulose have been tested for the removal of viruses (e.g., xenotropic murine and swine influenza viruses) and also of Au NPs from aqueous solutions [232,233,234].

Bacterial cellulose-based membranes have been developed and their performance was compared with commercial membranes for water pollutants removal. BNC nano-paper sheets have been used as ultrafiltration membranes with more than 75% of rejection efficiency towards polyethylene glycol. BNC rejection efficiency can be further increased to 85 to 90% by lamination with β-chitin or deacetylated chitin sulfonate composites [236]. The combination of BNC membranes and cross-linked CNF offers high separation efficiency (>98%) of stabilized or non-stabilized oil-in-water emulsions, with droplet diameters below 1 µm [7]. Other filtration features for the removal of specific components can be added to nanocomposite BNC membranes. For instance, BNC and GO are combined into a nanocomposite membrane by diffusing the GO into a BNC formamide gel. The freestanding BNC/GO membranes display specific permeation for distinct inorganic/organic ions with variable sizes (from the nano-scale to molecular-scale ranges) [205], enhanced mechanical strength and toughness, and high stability against swelling or water environments. Table 7 provides an overview of the use of nanocelluloses for the fabrication of filtration membranes and their contribution to wastewater treatment. 

## 9. Water Disinfection Materials from Pathogenic Microorganisms 

Disinfection processes are the last and most important step in the wastewater treatment process, i.e., removal, deactivation or killing of pathogenic microorganisms. Over the past decades, several nanomaterials (metal, metal oxides, and polymer NPs) have been used as disinfectants to reduce the harmful disinfection by-products [251]. Nanocelluloses on their own have negligible effects on microbial inhibition, and they can serve as a carbon source for their development, especially bacteria, algae, and fungi. Therefore, antimicrobial and antibacterial activities should be added to nanocellulose based materials to inhibit microbe’s growth in contaminated water. In this aspect, antibacterial features can be added to nanocelluloses for instance by surface modification with antimicrobial polymers, metal NPs (e.g., Ag, Au), and metal oxide NPs (e.g., TiO_2_, CuO, ZnO, MgO) [252]. Surface modification of nanocelluloses with oxidized groups gives active sites (TEMPO oxidation) provides nanocelluloses with functionalized carboxyl groups with high affinity towards *E. coli* (rejection ~96–99%). The introduction of negative surface charges (carboxyl groups) offers resistance against Gram-positive and Gram-negative bacteria, and a high binding affinity to the negative charge near the bacterial membrane can be provided. The carboxyl groups on the nanocelluloses surface do not intrinsically act as antibacterial agents, but they can be loaded with appropriate antibacterial compounds. For instance, cationic surface modification by quaternary ammonium compounds, such as ammonium bromides [253], and ammonium chlorides [254], is a straightforward method to introduce antibacterial activity. 

**Figure 13:**
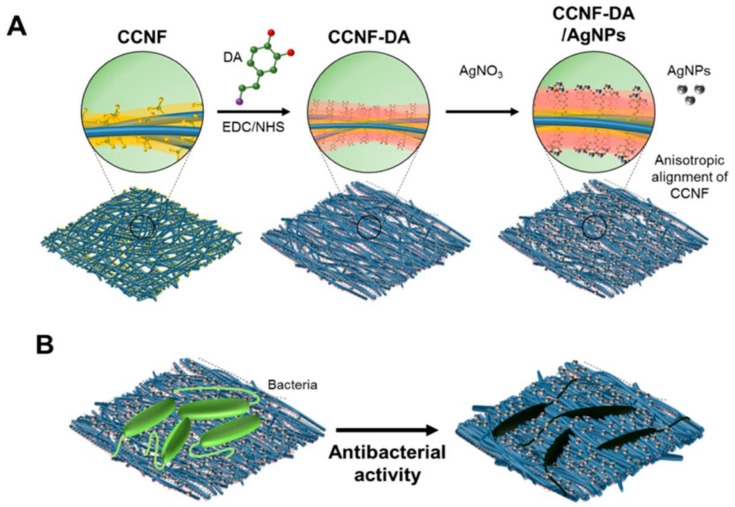
Schematic description of (**A**) anisotropic carboxylated cellulose nanofiber (CCNF)-dopamine (DA)/silver nanoparticle (Ag NPs) composite formation using 1-ethyl-3-(3-dimethylaminopropyl)carbodiimide (EDC) and N-hydroxysuccinimide (NHS); and (**B**) antibacterial activity of the CCNF-DA/Ag NP composite [258]. Reproduced with permission from Nguyen et al. (2016) © MDPI [Open access].

Nanocellulose also offers key functionalities that can be valued in antimicrobial filtration applications with antifouling properties. Nanocelluloses membranes with smaller pore sizes (~0.2 µm) in microfiltration membranes should allow the efficient elimination of bacteria (e.g., *E. coli*, *Salmonella*, *Hepatitis A* virus, *Cryptosporidium*). Mixtures of CNFs with cationically charged chitin nanofibrils allow the incorporation of antimicrobial activity while providing good stiffness and mechanical flexibility to the membranes [255]. In combination with the surface charge effect, the hydrophobicity variation through changes in the alkyl chain length enhances the compatibility with the lipid bilayer of the bacterial cells [256]. A study used antibacterial Ag NPs embedded in the matrix of an aluminum oxyhydroxide-nanocellulose composite to identify the optimum chloride concentration in water for effective antibacterial activity against *E. coli* and *B. subtilis*. The findings emphasized that increasing the chloride concentration drastically improves the antibacterial activity by reducing bacterial cell proliferation [257]. Recently, Nguyen et al, developed Ag NP-containing carboxylated CNF composites and dopamine (DA)-conjugated (CCNF-DA) with improved mechanical features, antimicrobial activity, and electrical conductivity by exploiting mussel’s utilization of catechols (Figure 13A). They found that the composite is anisotropically aligned due to the strong interaction between catechol moieties and Ag NPs, and that it endows electrically conductive biomaterials with flexibility, electronic conductivity, and antibacterial activity (Figure 13B). These materials may have a potential application as disinfectants for wastewater treatment. 

## 10. Current Challenges and Limitations

Even though cellulose-based composite materials show promising application in water purification for the elimination of heavy metal ions and organic dyes, it remains a big challenge to control the main factors of porosity, permeability, and specific adsorption. The control over the morphology of the membranes can be controlled through the presented methods in this review (e.g., electrospinning, coating, casting, freeze-drying), but a continuous and most cost-efficient industrial process for fabrication of the membranes still follows an easy papermaking-like route. However, urgent limitations to be resolved are attributed to the energetic efficiency for the formation of nanocellulose membranes (e.g., fabrication of nanofibrils, dewatering of the fiber mats), quality of the produced membranes (e.g., the occurrence of defects in the membranes and homogeneity in pore sizes), and performance (e.g., the relatively thick membranes are required to compensate for defects while decreasing the permeability and increasing pressure drops). In particular, the economic and cost-efficient upscaling of production of suitable membranes needs to be developed, in particular, controlling film formation and drying procedures to improve the morphology of high-flux membranes. The strong tendency for agglomeration of nanocellulose during film drying can be resolved by the deposition of nanocellulose onto given filters or membrane structures providing them with a thin functional layer.

In parallel, the viable surface modification will remain crucial to improving the selectivity and anti-fouling properties. The native nanocellulose membranes show moderate efficacy for adsorption and photocatalytic degradation, and several issues must be overcome to synthesize composite materials with adequate pollutant removal efficiency. Pollutant adsorption can occur through chemisorption or hydrogen bonding that can be facilitated by membrane surface functionalization with organic or inorganic materials. During adsorption, functional groups, such as −COOH and −OH, strongly interact with the pollutant molecules and have important roles in inorganic mineralization and the immobilization of metal oxides [120]. Therefore, alternative processing routes should be developed enhancing the reproducibility of single nanofiber geometries and acquiring more impact on their agglomeration within a web-like structure with controlled porosity and surface properties through a one-step processing route. A limited number of processing steps should therefore be foreseen, likely by introducing controllable charges at the nanofiber surface while avoiding traditional wet chemistry and using green solvents. 

The nanocellulose composite membranes have been proposed to increase the functionality (e.g., mechanical stability, chemical resistance, adsorption specificity, photodegradation) in particular for treatment of more complex water systems. A new aspect that will probably attract more attention in the future is the use of cellulose nanomaterials as the organic part in nanocomposites. The integrated cellulosic nanomaterial/inorganic material composites must be more thoroughly studied. More research is also required on cellulose/inorganic hybrids for the long-term treatment of natural water bodies and wastewater. However, the formation of cellulose/inorganic composite materials is a complex, not fully understood process. Several techniques have been used for the nanocomposite membrane synthesis, such as in situ precipitation [99].biomineralization [119], sol-gel [208], conventional casting [201], and co-precipitation [192]. The breakthrough of 3D printing can bring a more convenient method for the fabrication of complex nanocellulose composite membranes. Cellulose functionalization through oxidation or grafting improves its ability to initiate the growth of the inorganic nanoadsorbents and provide support for the immobilization of heavy metals [256]. One main challenge to the commercialization of nanocellulose composites is obtaining a homogenous mixture between cellulose and the inorganic nanoadsorbents. Indeed, homo-aggregation of the composite parts can negatively affect the morphology, efficiency and performance of the prepared composite materials [259]. Several techniques have been explored to address this issue, including the use of cellulose solvents [260], soluble cellulose derivatives [99], and inorganic mineralization control [261]. However, the regeneration and recyclability of the nanocomposite membranes should be considered from the design phase on. 

## 11. Conclusions

Developing eco-friendly water treatment materials with improved performance has received significant academic and industrial attention. To date, a large variety of approaches have been developed to address arguably one of the key challenges for humanity in the 21st century: clean water. Among the different nanomaterials, nanocelluloses are promising sustainable materials for wastewater treatment, in terms of performance and efficiency, and also lightweight with strong mechanical strength, chemical inertness, and versatile surface chemistry, inexpensive production costs, and safe handling compared with inorganic nanomaterials. The improvement of nanocellulose production has led many researchers to fabricate nanocellulose-based materials (individual NPs, hydrogels, aerogels, sponges, membranes) for wastewater treatment. Different nanocellulose forms (cellulose nanocrystals, cellulose fibers, bacterial cellulose) have been successfully investigated for water treatment, particularly in membranes and filters (size exclusion, e.g., for nanoparticle filtration, or affinity membranes) and also as adsorbents (e.g., heavy metal ions, dyes, drugs, pesticides, fertilizers). These nanocellulose-based materials show good scalability and regenerative capacity, as indicated by their good performance after several cycles (adsorption-desorption). Nanocellulose functionalization by surface grafting or blending with different inorganic materials, polymers, and carbon materials allow the mass production of new renewable and sustainable cellulose composites at a lower cost. The unique features of nanocelluloses and their high compatibility with many modifiers make them appropriate for water treatment by adsorption, absorption, photocatalytic degradation, disinfection, antifouling, ultrafiltration, nanofiltration and reverse osmosis. However, additional effort is required to implement and commercialize nanocelluloses as a viable nanomaterial for remediation technologies. Their dispersion stability and polydispersity, especially for industrial and commercial production, are another issue. The main challenge and limitations in working with nanocellulose-based materials must be identified to improve the development and efficient use of nanocelluloses in environmental remediation. The most effective strategy is to use nanocelluloses as support material for NP immobilization because NPs can freely penetrate the human body. However, impacts associated with a combination of nanocelluloses with functional NPs for use in wastewater treatment and the removal of NPs from the liquid after the reaction should be defined. This research field is rapidly growing and gearing towards the future commercial use of cellulose/inorganic hybrids for various industrial applications, including wastewater treatment. 

## Figures and Tables

**Table 1:** Classification of nanocellulose materials according to their morphology, origin, and mean size.

Type	Origin	Mean Size	Ref.
Cellulose Microfibers (CMF)	Wood, cotton, hemp, flax, straw	Several microns in diameter and hundred microns in length	[29]
Cellulose nanofiber (CNF)	Wood, cotton, hemp, flax, straw	Few microns in dimeter with finely attached nanofibers; several to hundred microns in length	[30]
Spherical cellulose nanoparticles (SCNPs)	Wood, cotton, hemp, flax, straw	Diameter: 10–100 nm	[31]
Cellulose nanocrystals (CNC)	Wood, cotton, hemp, flax, wheat straw, rice straw, mulberry bark, ramie, Avicel^®^, tunicin, algae	Width: 5–70 nm Length: 100–250 nm (from plants); 100 nm to several micrometers (from the cellulose of tunicates, algae, and bacteria)	[6]
Bacterial nanocellulose (BC)	Low molecular weight sugars and alcohols	Width: 5–70 nm Length: several micrometers	[32]
Cellulose nanoyarns (CNY)	Cellulose derivatives	Diameter; 100 to 1000 nm Length: >1 µm	[6]

**Table 3:** Nanocellulose materials and their surface modifications are exploited as adsorbents for heavy metal pollutant removal from wastewater. BC—bacterial cellulose.

Nanocellulose	Surface Modification	Heavy Metal	pH	Removal Efficiency (mg/g)	Langmuir			Freundlich			Ref.
					Q_max_ (mg·g^−1^)	K_L_ (L·mg^−1^)	R^2^	K_F_	1/n	R^2^	
Cellulose nanocrystals	Sulfate (-SO_3_^−^)	Ag^+^	6.39	34.35	1.9	0.09	0.996	˗	˗	˗	[60]
			3.5–4.5	56	˗	˗	˗	˗	˗	˗	[61]
		Cd^2+^	6.0	8	1.9	0.09	0.996	˗	˗	˗	[62]
			6.5	9.7	11.2	0.63	0.95	5.75	4.71	0.92	[63]
		Cu^2+^	3.5–4.5	19	˗	˗	˗	˗	˗	˗	[61]
			6	0.59	˗	˗	˗	˗	˗	˗	[64]
		Pb^2+^	5.5	47	27.9	0.04	0.973	˗	˗	˗	[62]
			6.5	9.42	10.20	1.81	0.98	7.06	6.89	0.94	[63]
		Fe^3+^	3.5–4.5	6.3	˗	˗	˗	˗	˗	˗	[61]
		Cr^3+^	6.5	1.6	˗	˗	˗	˗	˗	˗	[65]
		Cr^4+^	2.5	0.6	˗	˗	˗	˗	˗	˗	[65]
		Ni^2+^	6.5	8.55	11.23	0.33	0.97	3.91	2.90	0.97	[63]
		As^3+^	7.5	2.2	˗	˗	˗	˗	˗	˗	[66]
		As^4+^	2.5	2.3	˗	˗	˗	˗	˗	˗	[66]
	Phosphorylation (-PO_3_^2−^)	Ag^+^	3.5–4.5	106	˗	˗	˗	˗	˗	˗	[61]
		Cu^2+^	3.5–4.5	117	˗	˗	˗	˗	˗	˗	[61]
		Fe^3+^	3.5–4.5	114	˗	˗	˗	˗	˗	˗	[61]
	Succination (-COO^−^)	Cd^2+^	6	152	259.7	2.29	0.998	˗	˗	˗	[62]
		Pb^2+^	5.5	300	367.6	1.81	0.998	˗	˗	˗	[62]
		Cr^3+^	6.5	2.4	2.88	1.49	0.987	0.83	0.17	0.98	[65]
	Sodium salt succination (-COO^−^Na^+^)	Cd^2+^	6	167	344.8	41.88	1.000	˗	˗	˗	[62]
		Pb^2+^	5.5	300	465.1	4.13	1.000	˗	˗	˗	[62]
	Poly(itaconic acid/methacrylic acid)-grafted CNC/nanobentonite composite	Co^2+^	6	-	241.8	0.025	0.99	16.31	0.504	0.98	[67]
	Xanthate (-ROCS2^−^Na^+^)	Cd^2+^	6.0	24.3	154.3	0.11	0.99	26.9	2.38	0.98	[68]
	TEMPO-oxidation (-COO^−^)	Cu^2+^	6	14.6	˗	˗	˗	˗	˗	˗	[64]
	Periodate/chlorite oxidation	Cu^2+^	4	185	˗	˗	˗	˗	˗	˗	[69]
	Itaconic acid-grafted-magnetite modified with 2-mercaptobenzamide	Hg^2+^	8	69.7	˗	˗	˗	44.05	0.36	0.991	[70]
	Carboxylation (-COO^−^)	Pb^2+^	7	˗	2270	˗	0.978	˗	˗	˗	[71]
	Amination (NH_3_^+^)	Cr^6+^	2.5	9.8	2.77	0.48	0.968	0.58	0.08	0.96	[65]
		As^3+^	7.5	9.3	10.56	1.85	0.991	5.52	0.22	0.986	[66]
		As^4+^	2.5	9.8	12.06	5.34	0.992	6.13	0.75	0.941	[66]
	Carboxylated CNC/alginate composite	Pb^2+^	5.2	280	339.0	0.17	0.993	68.93	0.31	0.607	[72]
Cellulose nanofibers	Unmodified	Ag^+^	5.45	15.45	˗	˗	˗	˗	˗	˗	[60]
		Cu^2+^	6.2	13	-	˗	˗	˗	˗	˗	[58]
			4.5	17.9	24.8	0.327	0.969	0.04	0.69	0.992	[73]
		Pb^2+^	5	˗	16.5	49.14	0.64	2.9	1.48	0.74	[59]
		Zn^2+^	6	3.5	˗	˗	˗	˗	˗	˗	[58]
		Cr^3+^	5	14.1	˗	˗	˗	˗	˗	˗	[58]
	Phosphorylation (-PO_3_^2−^)	Ag^+^	3.5–4.5	120	˗	˗	˗	˗	˗	˗	[61]
		Cu^2+^	3.5–4.5	114	˗	˗	˗	˗	˗	˗	[61]
		Fe^3+^	3.5–4.5	73	˗	˗	˗	˗	˗	˗	[61]
	TEMPO-oxidation (-COO^−^)	Cu^2+^	6	112	˗	˗	˗	˗	˗	˗	[74]
			6.2	112	˗	˗	˗	˗	˗	˗	[74]
			5	˗	18.9	0.17	0.975	6.71	0.24	0.903	[72]
			5.7	114	˗	˗	˗	˗	˗	˗	[75]
		Ni^2+^	6	8.6	˗	˗	˗	˗	˗	˗	[74]
			6	49	˗	˗	˗	˗	˗	˗	[58]
		UO_2_^2+^	6.5	167	˗	˗	˗	˗	˗	˗	[56]
		Pb^2+^	6	9.7	˗	˗	˗	˗	˗	˗	[74]
		Zn^2+^	6.2	66.0	˗	˗	˗	˗	˗	˗	[74]
			6	66	˗	˗	˗	˗	˗	˗	[58]
		Cr^3+^	6	8.9	˗	˗	˗	˗	˗	˗	[74]
			5	58	˗	˗	˗	˗	˗	˗	[58]
	Mercerization/Succination (-COO^−^)	Cd^2+^	5	5	2.06	691	0.923	˗	˗	˗	[76]
		Co^2+^	5	5	1.34	3.55	0.984	˗	˗	˗	[76]
		Cu^2+^	5	4.7	1.90	58.89	0.849	˗	˗	˗	[76]
		Ni^2+^	5	4.6	0.74	12.32	0.964	˗	˗	˗	[76]
		Zn^2+^	5	5	1.61	59.21	0.902	˗	˗	˗	[76]
	Amination (NH_3_^+^)	Cd^2+^	5	58.1	405.6	61.30	0.892	˗	˗	˗	[77]
		Cu^2+^	5	50.6	195.6	4.072	0.770	˗	˗	˗	[77]
	Poly (acrylic acid) grafting	Cu^2+^	4.5	45.8	57.5	0.124	0.972	0.104	0.720	0.988	[73]
	Poly (acrylic acid)/sodium humate grafting	Cu^2+^	4.5	44.7	64.6	0.175	0.970	0.11	0.66	0.991	[73]
	TEMPO oxidation/polyethyleneimine grafting	Cu^2+^	5	˗	52.3	0.17	0.985	31.0	10.4	0.919	[78]
	TEMPO-oxidation/GO nanocomposite	Cu^2+^	5.7	63.5	˗	˗	˗	˗	˗	˗	[79]
	TEMPO-oxidation/nanoGO composite	Cu^2+^	5.7	68.1	˗	˗	˗	˗	˗	˗	[79]
	TEMPO-oxidation (-COO^−^)/thiolation (-Si-SH)	Hg^2+^	5–9	145	729.9	0.36	0.998	112.0	0.43	0.835	[78]
		Pb^2+^	5.5	133	137.7	0.783	0.998	˗	˗	˗	[80]
		Cr^6+^	4	76.5	87.5	0.308	0.997	˗	˗	˗	[80]
	Poly-methacylic acid-co-maleic acid grafting	Pb^2+^	5	165	˗	˗	˗	˗	˗	˗	[81]
		Ni^2+^	5	117	˗	˗	˗	˗	˗	˗	[81]
		Zn^2+^	5	138	˗	˗	˗	˗	˗	˗	[81]
		Cd^2+^	5	135	˗	˗	˗	˗	˗	˗	[81]
	Bisphosphonate (-PO(OH)_2_)_2_	V^5+^	3	10.3	1.13–1.98	1.69–2.92	0.97–0.996	0.65–1.44	0.41–0.48	0.87–0.993	[82]
	Sulfonation	Pb^2+^	5	123	251	0.69	0.97	72.8	0.66	0.93	[59]
	Periodate/chlorite oxidation/polyamide-amine-epichlorohydrin	Cu^2+^	-	82.19	-	-	-	-	-	-	[83]
		Pb^2+^		76.11	-	-	-	-	-	-	[83]
		Ca^2+^	-	94.77	-	-	-	-	-	-	[83]
Bacterial nanocellulose	Unmodified	Cu^2+^	4.5	9.67	11.2	0.011	0.962	5.389	0.13	0.838	[84]
			˗	˗	90.91	0.002	0.876	1.626	0.53	0.909	[85]
		Pb^2+^	4.5	22.6	24.59	0.084	0.976	5.17	0.30	0.960	[84]
			˗	˗	100.0	0.004	0.799	1.37	0.64	0.802	[85]
	Phosphorylation (-PO_3_^2−^)	Co^2+^	4.5	4.25	˗	˗	˗	˗	˗	˗	[86]
		Cu^2+^	4.5	4.77	˗	˗	˗	˗	˗	˗	[86]
		Mn^2+^	4.5	3.92	˗	˗	˗	˗	˗	˗	[86]
		Zn^2+^	4.5	4.36	˗	˗	˗	˗	˗	˗	[86]
		Fe^3+^	4.5	4.19	˗	˗	˗	˗	˗	˗	[86]
		Ho^3+^	8	13.6	˗	˗	˗	˗	˗	˗	[86]
		La^3+^	8	10.9	12.6	˗	˗	˗	˗	˗	[86]
		Sm^3+^	8	12.4	˗	˗	˗	˗	˗	˗	[86]
	Amination (NH_3_^+^)	Pb^2+^	4.5	57.2	87.41	0.003	0.993	0.995	0.62	0.982	[87]
	Carboxymethylation (-COO^−^)	Pb^2+^	4.5	60.4	65.53	0.058	0.961	5.70	0.50	0.898	[84]
	Fe_3_O_4_/BC hybrid nanocomposite	Pb^2+^	7	65	˗	˗	˗	˗	˗	˗	[75]
	Polyethyleneimine grafting	Pb^2+^	5.5	28.6	125.0	0.002	0.500	1.10	0.66	0.793	[85]
	Fe_3_O_4_/BC hybrid nanocomposite	Cr^3+^	7	25	˗	˗	˗	˗	˗	˗	[75]
		Mn^2+^	7	33	˗	˗	˗	˗	˗	˗	[75]

**Table 4:** Overview of the different nanocellulose types and their applications as adsorbents to remove organic pollutants.

Nanocellulose	Surface Modification	Dye	pH	Removal Efficiency mg/g	Langmuir			Freundlich			Ref.
					Q_max_ (mg·g^−1^)	K_L_ (L·mg^−1^)	R^2^	K_F_	1/n	R^2^	
Cellulose nanocrystals	Sulfate (-SO_3_^−^)	Basic fuchsin	6–9	261	˗	˗	˗	˗	˗	˗	[118]
		Crystal violet	6–9	178	185	1.023	0.997	44.7	0.26	0.68	[127]
		Methylene blue	9	-	118	0.014	0.99	18.56	0.27	0.97	[118]
			6–9	178	˗	˗	˗	˗	˗	˗	[118]
			7	107	˗	˗	˗	˗	˗	˗	[128]
		Malachite green	6–9	92	˗	˗	˗	˗	˗	˗	[118]
	Carboxylation (-COO^−^)	Basic fuchsin	6–9	318	˗	˗	˗	˗	˗	˗	[118]
		Crystal violet	6–9	223	243.9	1.129	0.986	76.50	0.21	0.803	[118]
		Methylene blue	9	-	769	0.009	0.99	˗	˗	˗	[128]
			6–9	233	˗	˗	˗	˗	˗	˗	[118]
			7	79	101.2	0.019	0.974	˗	˗	˗	[129]
		Malachite green	6–9	148	˗	˗	˗	˗	˗	˗	[118]
	Amination (NH_3_^+^)	Acid red GR	3.5	199.9	873.8	0.0305	0.92	120.3	0.34	0.985	[130]
		Congo red	4.7	199.5	˗	˗	˗	˗	˗	˗	[131]
		Light yellow K-4G	4.7	183.0	˗	˗	˗	˗	˗	˗	[131]
	PVAm grafting	Acid red GR	3.5	199.9	873.8	0.0305	0.92	120.3	0.34	0.985	[130]
		Light yellow K-4G	3.5	196.9	1211	0.046	0.80	237.1	0.26	0.974	[130]
		Congo red	3.5	199.8	1619.9	0.210	0.96	267.3	0.56	0.770	[130]
	CNC-infused polyacrylonitrile membrane	Crystal violet	7	4	68	˗	0.993	˗	˗	˗	[132]
	Keratin composite	Direct Red 80	2	485	1111	0.043	0.995	186.8	0.28	0.944	[133]
		Reactive Black 5	2	475	1250	0.028	0.992	156.8	0.33	0.976	[133]
	Alginate hydrogel	Methylene blue	7	˗	256.4	0.002	0.998	1.931	0.649	0.988	[134]
	Polyacrylamide composite	Methylene blue	6.5	19	˗	˗	˗	˗	˗	˗	[135]
	Chitosan-g-poly(acrylic acid) composite (5 wt% CNC)	Methylene blue	6	1968	2074	0.139	0.999	˗	˗	˗	[136]
	Imidazolium grafting	Orange II	-	98	˗	˗	˗	˗	˗	˗	[137]
	UnmodifiedQuaternization	Methylene blue	9	100	122.2	0.178	0.996	12.8	0.22	0.85	[138]
		Crystal violet	-	664	˗	˗	˗	˗	˗	˗	[139]
Cellulose nanofibers	Unmodified	Acid green 25	-	683	˗	˗	˗	˗	˗	˗	[139]
	TEMPO-oxidation (-COO^−^) TEMPO-periodate-chlorite oxidation	Methylene blue	7	-	˗	˗	˗	˗	˗	˗	[96]
		Methylene blue	7	-	502	˗	˗	64	˗	˗	[96]

**Table 6:** Nanocellulose/inorganic composites as effective wastewater cleaning agents.

Cellulosic Material	Inorganic	Preparation Method	Removal Process	Pollutants	Removal Capacity	References
Cellulose-graft-soy protein isolate	Calcium phosphate–rod-like shape hydroxyapatite	Bio mineralization	Adsorption	Methylene blue	454 mg/g	[161]
Carboxymethyl cellulose	Hydroxyapatite	In situ precipitation	Adsorption	Pb^2+^ acid yellow 220	625 mg/g and 200 mg/g	[97]
Carboxymethyl cellulose-graft-polymethacrylic acid	Rod-like hydroxyapatite	Bio mineralization	Adsorption	Methylene blue	671 mg/g	[121]
Carboxymethyl cellulose	Carboxymethyl cellulose, hydroxyapatite and lysine membrane	Conventional casting or double decomposition methods	Adsorption	Bisphenol A		[201]
Cellulose	Montmorillonite	-	Adsorption	Cr (VI)	22.2 mg/g	[21]
Cellulose acetate	Organically modified montmorillonite	-	Adsorption	Cr (VI)	-	[98]
Cellulose and carboxymethyl cellulose	Clay	Crosslinking in NaOH/urea aqueous solution	Adsorption	Methylene blue	98%	[202]
Cellulose	GO/cellulose membranes	Pressed membranes	Adsorption	Heavy metals	-	[203]
Dialdehyde cellulose	GO	Crosslinked with triethylenetetramine	Adsorption	Cu^2+^ and Pb^2+^	65.1 and 80.9 mg/g	[102]
MCC	GO	GO/microcrystalline cellulose aerogels prepared using lithium bromide	Adsorption	Methylene blue	2630 mg/g	[204]
CNC	rGO	3D sponge using vitamin C	Adsorption	Methylene blue	850 mg/g	[160]
Cellulose	GO	Wet-spinning technique	Adsorption	Methylene blue	481mg/g	[161]
Bacterial cellulose	GO	Composite membrane	Selective ion permeation	Different inorganic/organic ions	-	[205]
Cellulose	GO	Using ionic liquids as solvent	Adsorption	Ce^3+^	109.1 mg/g	[205]
Cellulose	CNTs	Grafting Welan gum onto CNTs entrapped cellulose beads	Adsorption	Methylene blue	300 mg/g	[206]
CNCs	Carbon monolith	-	Adsorption	Methylene blue	127 mg/g	[129]
Bacterial cellulose	Fe_3_O_4_	Cellulose-magnetite composites	Adsorption	Cr(VI)	---	[104]
Bacterial cellulose	Fe_3_O_4_	Agitation fermentation	Adsorption	Pb^2+^, Mn^2+^ and Cr^3+^	65, 33 and 25 mg/g	[75]
Mesquite tree pulp	Fe_3_O_4_	Spherical magnetic NPs through co-precipitation	Adsorption	Methylene blue	1225 mg/g	[120]
Cellulose acetate fibers	Zno NPs with Fe_3_O_4_	Coprecipitation	Adsorption	Phenol	64%	[157]
Carbon microspheres	Magnetic CoFe_2_O_4_	-	Photodegradation	Rhodamine B	-	[194]
Cellulose	Fe_3_O_4_	Co-precipitation	Photodegradation	Methylene blue	-	[192]
Cellulose	Fe_3_O_4_	Hydrothermal technique	Photodegradation	Rhodamine B	100%	[196]
Carboxyl cellulose	Fe_3_O_4_	Chemical co-precipitation technique	Photodegradation	Navy blue	-	[207]
Amidoximated bacterial cellulose	ZnO	*In situ* polyol method	Photocatalytic property	Methyl orange	92%	[184]
Cellulosic fabric	ZnO	Sol-gel process	Photodegradation	Methylene blue	-	[208]
Cellulose	ZnO	Co-precipitation	Photodegradation	Methylene blue	69.5%	[209]
Cellulose	TiO_2_	Acids	Photodegradation	-	-	[210]
Hydroxypropyl methyl cellulose	TiO_2_	Nano-photocatalysts prepared in situ	Photodegradation	4-nitrophenol		[211]
Bacterial cellulose	TiO_2_	-	-	Pb	90%	[212]
CMC	TiO_2_	Polyaniline/CMC/TiO_2_ nanocomposite	Adsorption	Congo red	94.3 mg/g	[213]
Paper	TiO_2_	-	Catalytic degradation	Acetaldehyde	-	[214]
Regenerated Cellulose	TiO_2_	Sol–gel	Photodegradation	Phenol		[195]
Cellulose	BiOBr	-	Photo-degradation	Phenol	80%	[198]
Nanocellulose	Ag	In situ precipitation	Adsorption	Pb^2+^ Cr^3+^ and	99.5%, and 98.3%	[215]

**Table 7:** Filtration membranes are made of nanocelluloses for wastewater treatment. CA, cellulose acetate; TMP, transmembrane pressure; PAE, polyamideamine-epichlorohydrin; PAN, polyacrylonitrile; PET, polyethylene terephthalate.

Nano Celluloses	Membrane	Pollutants	Thickness (µm)	Porosity (%)	Flux (L.h^−1^.m^−2^)	TMP (kPa)	Permeability (L.h^−1^.m^−2^.Pa^−1^)	Efficiency (% or mg·g^−1^)	Ref.
Cellulose nanocrystals	Unmodified CNC	PEG	20–60	35	-	200–500	1.8 × 10^−5^–6.1 × 10^−5^	20–75%	[237]
		Oil	0.6–10	-	1036–1734	50	2.1 × 10^−2^–3.5 × 10^−2^	99.9%	[238]
	CNC-coated CNF	Ag^+^ Cu^2+^ Fe^2+^ Fe^3+^	201–210	22–23	0	450	0	91–94%	[239]
	Acetone treated CNC-coated CNF		201–210	29–34	8.8–13.9	450	2.0 × 10^−5^–3.1 × 10^−5^	-	[239]
	Oxidized CNC/gelatin-coated cellulose sludge/CNF		166–208	-	-	450	7.7 × 10^−5^–5.5 × 10^−4^	0.81–0.87 mg·g^−1^	[240]
	CNC/gelatin-coated cellulose		440–448	65	-	50–150	9.5 × 10^−3^	0.33 mg·g^−1^	[241]
	Phosphorylated CNC/gelatin-coated cellulose		440–448	65	-	50–150	2.6 × 10^−2^	0.33 mg·g^−1^	[241]
	CNC/chitosan	Methyl violet 2B	250–270	-	-	196	6.4 × 10^−5^	90%	[242]
	Cross-linked CNC/chitosan		250–270	-	-	196	6.4 × 10^−5^	48–91%	[242]
	CNC/chitosan	Rhodamine 6G	250–270	-	-	196	6.4 × 10^−5^	69%	[242]
	Cross-linked CNC/chitosan		250–270	-	-	196	6.4 × 10^−5^	13–70%	[242]
	CNC-impregnated electrospun CA	Victoria blue 2B	52–116	69–73	17,838	600–800	2.069 × 10^−2^	52.6–99.9%	[243]
	CNC/chitosan		250–270	-	-	196	6.4 × 10^−5^	98%	[242]
	Cross-linked CNC/chitosan		250–270	-	-	196	6.4 × 10^−5^	88–98%	[242]
	Oxidized CNC-impregnated electrospun PAN	Phage MS2	45	80	192	30	6.4 × 10^−2^	99%	[133]
	CNC-impregnated electrospun CA		52–116	69–73	17,838	600–800	2.069 × 10^−2^	2.5–25.9%	[243]
Cellulose nanofibers	Unmodified CNF	Au	0.496–0.564	55.8–68.5	0.952–2.2	-	1.19 × 10^−5^–2.75 × 10^−5^	84.6–93.5%	[244]
		Ferritin (12 nm)	0.496–0.564	55.8–68.5	0.952–2.2	-	1.19 × 10^−5^–2.75 × 10^−5^	90.2–94.3%	[244]
		Polyethylene glycol	25–65	35	-	200–10^3^	10^−6^–4 × 10^−6^	88–100%	[237]
			20–70	35	-	200–10^3^	4 × 10^−6^–2.7 × 10^−5^	88–100%	[80]
		Polystyrene	20–70	35	-	200–10^3^	4 × 10^−6^–2.7 × 10^−5^	75–100%	[80]
		Ag^+^	176	23–25	0–2.9	450	0–6.4 × 10^−6^	77%	[239]
		Cu^2+^	176	23–25	0–2.9	450	0–6.4 × 10^−6^	94%	[239]
		Methylene blue	0.496–0.564	55.8–68.5	0.952–2.2		1.19 × 10^−5^–2.75 × 10^−5^	99.22%	[244]
	Oxidized CNF	Polyethylene glycol	20–70	35	-	200–10^3^	10^−6^–4 × 10^−6^	58–100%	[237]
		Polyethylene glycol Polystyrene	20–70	35	-	200–10^3^	1.2 × 10^−6^–5 × 10^−6^	58–95%	[80]
			20–70	35	-	200–10^3^	1.2 × 10^−6^–5 × 10^−6^	67–100%	[80]
		Ca^2+^/SO_4_^2−^	20–70	35	-	200–10^3^	10^−6^–4 × 10^−6^	5–34%	[245]
		Oil	20–100	-	192	10^3^	1.92 × 10^−4^	92.9–97.9%	[217]
	PAE cross-linked CNF	Polyethylene glycol	20	-	110	200	5.5 × 10^−4^	-	[246]
	CNF/silica/PAE	White water effluent	0.55–0.64	-	98–122	500–10^3^	1.2 × 10^−4^–2.0 × 10^−4^	93%	[247]
	PAE cross-linked oxidized CNF	Ag(I)	176	34–42	6.3–8.7	450	1.4 × 10^−5^–1.9 × 10^−5^	-	[239]
	Acetone treated CNF	Cr(VI)	200	78	-	-	0.145	60%	[222]
	Thiol-functionalized CNF- impregnated electrospun PAN	Cr(VI) Cu^2+^	40	80	-	-	0.189	100%	[247]
	Aminated CNF-impregnated electrospun PAN/PET		25	-	-	200	2.3 × 10^−6^	7.7%	[245]
	Phosphorylated CNF	Cu^2+^ Pb^2+^	176	34–42	6.3–8.7	450	1.4 × 10^−5^–1.9 × 10^−5^	-	[239]
	Acetone-treated CNF		200	78	-	-	0.145	137.7 mg·g^−1^	[222]
	Thiol-functionalized CNF- impregnated electrospun PAN	Pb^2+^ Br^−^	40	80	215	-	0.189	260 mg·g^−1^	[247]
	Aminated CNF-impregnated electrospun PAN/PET		40 -	80 -	215 215	- -	0.189 -	260 mg·g^−1^ 89.6 mg·g^−1^	[247] [248]
	Oxidized CNF-coated Ti-Bi oxide								
		Cs^+^	-	-	215	-	-	125.4 mg·g^−1^	[248]
		I^−^	-	-	215	-	-	225.9 mg·g^−1^	[248]
		SeO_3_^2−^	-	-	215	-	-	204.5 mg·g^−1^	[248]
		SeO_4_^2−^	-	-	215	-	-	85.1 mg·g^−1^	[248]
		Sr^2+^	-	-	215	-	-	81.4 mg·g^−1^	[248]
		Polyethylene glycol	16	-	18–54	196–392	6.9 × 10^−5^–2.8 × 10^−4^	30–50%	[249]
Bacterial cellulose (BC)	Unmodified BC	Polyethylene glycol Oil	15–85	35	-	200–10^3^	5 × 10^−6^–2 × 10^−5^	60–75%	[237]
			20–100	95–97	441–845	10^3^	4.41 × 10^−4^–8.45 × 10^−4^	98.3–99.3%	[7]
		*Chlorella* sp.	8–27	1.4–2.4	3.2–11.7	0–220	3.2 × 10^−5^–1.17 × 10^−4^	99.6–99.8%	[249]
		Bovine serum albumin	8–27	1.4–2.4	11–81	0–220	1.1 × 10^−4^–8.1 × 10^−4^	61.6–99.0%	[249]
		Polyethylene glycol	16–102	-	0.03–6	196–392	7.6 × 10^−8^ -1.5 × 10^−5^	90%	[236]
	BC derivative	Polyethylene glycol Inorganic/organic ions	16–102	-	8–15	196–392	5 × 10^−6^–5 × 10^−5^	85–90%	[236]
	Chitin laminated BC		11–17	-	-	-	-	-	[205]
	GO/BC	Reactive red X-3B dye	14	-	1146	-	-	-	[250]
	Cobalt-phthalocyanine immobilized BC								

## Data Availability

The data presented in this study are available on request from the corresponding author.

## References

[1] Ranade V.V., Bhandari V.M. (2014). Industrial Wastewater Treatment, Recycling, and Reuse: An Overview. Industrial Wastewater Treatment, Recycling and Reuse.

[2] Wiesmann U., Choi I.S., Dombrowski E.M. (2006). Fundamentals of Biological Wastewater Treatment.

[3] Ahankari S., George T., Subhedar A., Kar K.K. (2020). Nanocellulose as a sustainable material for water purification. SPE Polym..

[4] Barhoum A., Jeevanandam J., Rastogi A., Samyn P., Boluk Y., Dufresne A., Danquah M.K., Bechelany M. (2020). Plant celluloses, hemicelluloses, lignins, and volatile oils for the synthesis of nanoparticles and nanostructured materials. Nanoscale.

[5] Barhoum A., Li H., Chen M., Cheng L., Yang W., Dufresne A. (2019). Emerging Applications of Cellulose Nanofibers. Handbook of Nanofibers.

[6] Trache D., Tarchoun A.F., Derradji M., Hamidon T.S., Masruchin N., Brosse N., Hussin M.H. (2020). Nanocellulose: From Fundamentals to Advanced Applications. Front. Chem..

[7] Hassan E., Hassan M., Abou-Zeid R., Berglund L., Oksman K. (2017). Use of Bacterial Cellulose and Crosslinked Cellulose Nanofibers Membranes for Removal of Oil from Oil-in-Water Emulsions. Polymers.

[8] Nnaji C.O., Jeevanandam J., Chan Y.S., Danquah M.K., Pan S., Barhoum A. (2018). Engineered nanomaterials for wastewater treatment: Current and future trends. Fundamentals of Nanoparticles.

[9] Hassan M.L., Fadel S.M., Abouzeid R.E., Elseoud W.S.A., Hassan E.A., Berglund L., Oksman K. (2020). Water purification ultrafiltration membranes using nanofibers from unbleached and bleached rice straw. Sci. Rep..

[10] Mahfoudhi N., Boufi S. (2017). Nanocellulose as a novel nanostructured adsorbent for environmental remediation: A review. Cellulose.

[11] Mautner A. (2020). Nanocellulose water treatment membranes and filters: A review. Polym. Int..

[12] Hassan M., Abou-Zeid R., Hassan E., Berglund L., Aitomäki Y., Oksman K. (2017). Membranes Based on Cellulose Nanofibers and Activated Carbon for Removal of Escherichia coli Bacteria from Water. Polymers.

[13] Hassan M., Hassan E., Fadel S.M., Abou-Zeid R.E., Berglund L., Oksman K. (2018). Metallo-Terpyridine-Modified Cellulose Nanofiber Membranes for Papermaking Wastewater Purification. J. Inorg. Organomet. Polym. Mater..

[14] Mohammed N., Grishkewich N., Tam K.C. (2018). Cellulose nanomaterials: Promising sustainable nanomaterials for application in water/wastewater treatment processes. Environ. Sci. Nano.

[15] Shak K.P.Y., Pang Y.L., Mah S.K. (2018). Nanocellulose: Recent advances and its prospects in environmental remediation. Beilstein J. Nanotechnol..

[16] Zhang K., Barhoum A., Xiaoqing C., Li H., Samyn P. (2019). Cellulose Nanofibers: Fabrication and Surface Functionalization Techniques. Handbook of Nanofibers.

[17] Li R., Zhang L., Wang P. (2015). Rational design of nanomaterials for water treatment. Nanoscale.

[18] El-Gendy A., Abou-Zeid R.E., Salama A., Diab M., El-Sakhawy M. (2017). TEMPO-oxidized cellulose nanofibers/polylactic acid/TiO_2_ as antibacterial bionanocomposite for active packaging. Egypt. J. Chem..

[19] Abouzeid R.E., Khiari R., Salama A., Diab M., Beneventi D., Dufresne A. (2020). In situ mineralization of nano-hydroxyapatite on bifunctional cellulose nanofiber/polyvinyl alcohol/sodium alginate hydrogel using 3D printing. Int. J. Biol. Macromol..

[20] Hassan M., Berglund L., Hassan E., Abou-Zeid R., Oksman K. (2018). Effect of xylanase pretreatment of rice straw unbleached soda and neutral sulfite pulps on isolation of nanofibers and their properties. Cellulose.

[21] Ho T.T.T., Zimmermann T., Hauert R., Caseri W. (2011). Preparation and characterization of cationic nanofibrillated cellulose from etherification and high-shear disintegration processes. Cellulose.

[22] Chen Y., He Y., Fan D., Han Y., Li G., Wang S. (2017). An Efficient Method for Cellulose Nanofibrils Length Shearing via Environmentally Friendly Mixed Cellulase Pretreatment. J. Nanomater..

[23] Zhang J., Elder T.J., Pu Y., Ragauskas A.J. (2007). Facile synthesis of spherical cellulose nanoparticles. Carbohydr. Polym..

[24] Dai J., Chae M., Beyene D., Danumah C., Tosto F., Bressler D.C. (2018). Co-Production of Cellulose Nanocrystals and Fermentable Sugars Assisted by Endoglucanase Treatment of Wood Pulp. Materials.

[25] Torres F., Troncoso O., López D., Grande C., Gómez C.M. (2009). Reversible stress softening and stress recovery of cellulose networks. Soft Matter.

[26] Mittal N., Ansari F., Gowda Krishne V., Brouzet C., Chen P., Larsson P.T., Roth S.V., Lundell F., Wågberg L., Kotov N.A. (2018). Multiscale Control of Nanocellulose Assembly: Transferring Remarkable Nanoscale Fibril Mechanics to Macroscale Fibers. ACS Nano.

[27] Rodríguez K., Sundberg J., Gatenholm P., Renneckar S. (2014). Electrospun nanofibrous cellulose scaffolds with controlled microarchitecture. Carbohydr. Polym..

[28] Rege A., Preibisch I., Schestakow M., Ganesan K., Gurikov P., Milow B., Smirnova I., Itskov M. (2018). Correlating Synthesis Parameters to Morphological Entities: Predictive Modeling of Biopolymer Aerogels. Materials.

[29] Siró I., Plackett D., Hedenqvist M., Ankerfors M., Lindström T. (2011). Highly transparent films from carboxymethylated microfibrillated cellulose: The effect of multiple homogenization steps on key properties. J. Appl. Polym. Sci..

[30] Lindström T. (2017). Aspects on nanofibrillated cellulose (NFC) processing, rheology and NFC-film properties. Curr. Opin. Colloid Interface Sci..

[31] Lin C., Ma Q., Su Q., Bian H., Zhu J.Y. (2018). Facile Synthesis of Highly Hydrophobic Cellulose Nanoparticles through Post-Esterification Microfluidization. Fibers.

[32] Zhong C. (2020). Industrial-Scale Production and Applications of Bacterial Cellulose. Front. Bioeng. Biotechnol..

[33] Barhoum A., Pal K., Rahier H., Uludag H., Kim I.S., Bechelany M. (2019). Nanofibers as new-generation materials: From spinning and nano-spinning fabrication techniques to emerging applications. Appl. Mater. Today.

[34] Kumar A.S.K., Kalidhasan S., Rajesh V., Rajesh N. (2012). Application of Cellulose-Clay Composite Biosorbent toward the Effective Adsorption and Removal of Chromium from Industrial Wastewater. Ind. Eng. Chem. Res..

[35] Sakthivel T., Reid D.L., Goldstein I., Hench L., Seal S. (2013). Hydrophobic High Surface Area Zeolites Derived from Fly Ash for Oil Spill Remediation. Environ. Sci. Technol..

[36] Gong R., Ye J., Dai W., Yan X., Hu J., Hu X., Li S., Huang H. (2013). Adsorptive Removal of Methyl Orange and Methylene Blue from Aqueous Solution with Finger-Citron-Residue-Based Activated Carbon. Ind. Eng. Chem. Res..

[37] Monier M., Abdel-Latif D.A. (2013). Synthesis and characterization of ion-imprinted resin based on carboxymethyl cellulose for selective removal of UO_2_^2+^. Carbohydr. Polym..

[38] Salama A., Hesemann P. (2018). Synthesis of N-Guanidinium-Chitosan/Silica Hybrid Composites: Efficient Adsorbents for Anionic Pollutants. J. Polym. Environ..

[39] Salama A., Hesemann P. (2018). New N-guanidinium chitosan/silica ionic microhybrids as efficient adsorbent for dye removal from waste water. Int. J. Biol. Macromol..

[40] Hassan H., Salama A., El-Ziaty A.K., El-Sakhawy M. (2019). New chitosan/silica/zinc oxide nanocomposite as adsorbent for dye removal. Int. J. Biol. Macromol..

[41] Fabryanty R., Valencia C., Soetaredjo F.E., Putro J., Santoso S.P., Kurniawan A., Ju Y.-H., Ismadji S. (2017). Removal of crystal violet dye by adsorption using bentonite–alginate composite. J. Environ. Chem. Eng..

[42] Naseer A., Jamshaid A., Hamid A., Muhammad N., Ghauri M., Iqbal J., Rafiq S., Khuram S., Shah N.S. (2019). Lignin and Lignin Based Materials for the Removal of Heavy Metals from Waste Water-An Overview. Z. Phys. Chem..

[43] Barhoum A., Samyn P., Öhlund T., Dufresne A. (2017). Review of recent research on flexible multifunctional nanopapers. Nanoscale.

[44] Abou-Zeid R.E., Khiari R., Beneventi D., Dufresne A. (2018). Biomimetic Mineralization of Three-Dimensional Printed Alginate/TEMPO-Oxidized Cellulose Nanofibril Scaffolds for Bone Tissue Engineering. Biomacromolecules.

[45] Xu Q., Poggi G., Resta C., Baglioni M. (2020). Grafted nanocellulose and alkaline nanoparticles for the strengthening and deacidification of cellulosic artworks. J. Colloid Interface Sci..

[46] Rusli R., Eichhorn S. (2008). Determination of the stiffness of cellulose nanowhiskers and the fiber-matrix interface in a nanocomposite using Raman spectroscopy. Appl. Phys. Lett..

[47] Tanpichai S., Quero F., Nogi M., Yano H., Young R.J., Lindström T., Sampson W.W., Eichhorn S.J. (2012). Effective Young’s Modulus of Bacterial and Microfibrillated Cellulose Fibrils in Fibrous Networks. Biomacromolecules.

[48] Eyley S., Thielemans W. (2014). Surface modification of cellulose nanocrystals. Nanoscale.

[49] Habibi Y. (2014). Key advances in the chemical modification of nanocelluloses. Chem. Soc. Rev..

[50] Nguyen T., Roddick F.A., Fan L. (2012). Biofouling of Water Treatment Membranes: A Review of the Underlying Causes, Monitoring Techniques and Control Measures. Membranes.

[51] Abdelmouleh M., Boufi S., Belgacem M., Duarte A.P., Ben Salah A., Gandini A. (2004). Modification of cellulosic fibres with functionalised silanes: Development of surface properties. Int. J. Adhes. Adhes..

[52] Tan K.X., Barhoum A., Pan S., Danquah M.K. (2018). Risks and toxicity of nanoparticles and nanostructured materials. Emerging Applications of Nanoparticles and Architectural Nanostructures: Current Prospects and Future Trends.

[53] Wołowiec M., Komorowska-Kaufman M., Pruss A., Rzepa G., Bajda T. (2019). Removal of Heavy Metals and Metalloids from Water Using Drinking Water Treatment Residuals as Adsorbents: A Review. Minerals.

[54] El-Sayed M.E. (2020). Nanoadsorbents for water and wastewater remediation. Sci. Total Environ..

[55] Abou-Zeid R.E., Khiari R., El-Wakil N., Dufresne A. (2019). Current State and New Trends in the Use of Cellulose Nanomaterials for Wastewater Treatment. Biomacromolecules.

[56] Ma H., Hsiao B.S., Chu B. (2012). Ultrafine Cellulose Nanofibers as Efficient Adsorbents for Removal of UO_2_^2+^ in Water. ACS Macro Lett..

[57] Sirviö J.A., Ukkola J., Liimatainen H. (2019). Direct sulfation of cellulose fibers using a reactive deep eutectic solvent to produce highly charged cellulose nanofibers. Cellulose.

[58] Sehaqui H., De Larraya U.P., Liu P., Pfenninger N., Mathew A.P., Zimmermann T., Tingaut P. (2014). Enhancing adsorption of heavy metal ions onto biobased nanofibers from waste pulp residues for application in wastewater treatment. Cellulose.

[59] Suopajärvi T., Liimatainen H., Karjalainen M., Upola H., Niinimäki J. (2015). Lead adsorption with sulfonated wheat pulp nanocelluloses. J. Water Process. Eng..

[60] Liu P., Sehaqui H., Tingaut P., Wichser A., Oksman K., Mathew A.P. (2014). Cellulose and chitin nanomaterials for capturing silver ions (Ag^+^) from water via surface adsorption. Cellulose.

[61] Liu P., Borrell P.F., Božič M., Kokol V., Oksman K., Mathew A.P. (2015). Nanocelluloses and their phosphorylated derivatives for selective adsorption of Ag+, Cu2+ and Fe3+ from industrial effluents. J. Hazard. Mater..

[62] Yu X., Tong S., Ge M., Wu L., Zuo J., Cao C., Song W. (2013). Adsorption of heavy metal ions from aqueous solution by carboxylated cellulose nanocrystals. J. Environ. Sci..

[63] Kardam A., Raj K.R., Srivastava S., Srivastava M.M. (2014). Nanocellulose fibers for biosorption of cadmium, nickel, and lead ions from aqueous solution. Clean Technol. Environ. Policy.

[64] Hamid H.A., Jenidi Y., Thielemans W., Somerfield C., Gomes R.L. (2016). Predicting the capability of carboxylated cellulose nanowhiskers for the remediation of copper from water using response surface methodology (RSM) and artificial neural network (ANN) models. Ind. Crop. Prod..

[65] Singh K., Arora J.K., Sinha T.J.M., Srivastava S. (2014). Functionalization of nanocrystalline cellulose for decontamination of Cr(III) and Cr(VI) from aqueous system: Computational modeling approach. Clean Technol. Environ. Policy.

[66] Singh K., Sinha T., Srivastava S. (2015). Functionalized nanocrystalline cellulose: Smart biosorbent for decontamination of arsenic. Int. J. Miner. Process..

[67] Anirudhan T., Deepa J., Christa J. (2016). Nanocellulose/nanobentonite composite anchored with multi-carboxyl functional groups as an adsorbent for the effective removal of Cobalt(II) from nuclear industry wastewater samples. J. Colloid Interface Sci..

[68] Pillai S.S., Deepa B., Abraham E., Girija N., Geetha P., Jacob L., Koshy M. (2013). Biosorption of Cd(II) from aqueous solution using xanthated nano banana cellulose: Equilibrium and kinetic studies. Ecotoxicol. Environ. Saf..

[69] Sheikhi A., Safari S., Yang H., van de Ven T.G.M. (2015). Copper Removal Using Electrosterically Stabilized Nanocrystalline Cellulose. ACS Appl. Mater. Interfaces.

[70] Anirudhan T., Shainy F. (2015). Effective removal of mercury(II) ions from chlor-alkali industrial wastewater using 2-mercaptobenzamide modified itaconic acid-grafted-magnetite nanocellulose composite. J. Colloid Interface Sci..

[71] Sharma P.R., Chattopadhyay A., Zhan C., Sharma S.K., Geng L., Hsiao B.S. (2018). Lead removal from water using carboxycellulose nanofibers prepared by nitro-oxidation method. Cellulose.

[72] Zhang N., Zang G.-L., Shi C., Yu H.-Q., Sheng G.-P. (2016). A novel adsorbent TEMPO-mediated oxidized cellulose nanofibrils modified with PEI: Preparation, characterization, and application for Cu(II) removal. J. Hazard. Mater..

[73] Zhang X., Zhao J., Cheng L., Lu C., Wang Y., He X., Zhang W. (2014). Acrylic acid grafted and acrylic acid/sodium humate grafted bamboo cellulose nanofibers for Cu^2+^ adsorption. RSC Adv..

[74] Srivastava S., Kardam A., Raj K.R. (2012). Nanotech Reinforcement onto Cellulosic Fibers: Green Remediation of Toxic Metals. Int. J. Green Nanotechnol. Biomed..

[75] Zhu H., Jia S., Wan T., Jia Y., Yang H., Li J., Yan L., Zhong C. (2011). Biosynthesis of spherical Fe3O4/bacterial cellulose nanocomposites as adsorbents for heavy metal ions. Carbohydr. Polym..

[76] Hokkanen S., Repo E., Sillanpää M. (2013). Removal of heavy metals from aqueous solutions by succinic anhydride modified mercerized nanocellulose. Chem. Eng. J..

[77] Hokkanen S., Repo E., Suopajärvi T., Liimatainen H., Niinimaa J., Sillanpää M. (2014). Adsorption of Ni(II), Cu(II) and Cd(II) from aqueous solutions by amino modified nanostructured microfibrillated cellulose. Cellulose.

[78] Geng B., Wang H., Wu S., Ru J., Tong C., Chen Y., Liu H., Wu S., Liu X. (2017). Surface-Tailored Nanocellulose Aerogels with Thiol-Functional Moieties for Highly Efficient and Selective Removal of Hg(II) Ions from Water. ACS Sustain. Chem. Eng..

[79] Zhu C., Liu P., Mathew A.P. (2017). Self-Assembled TEMPO Cellulose Nanofibers: Graphene Oxide-Based Biohybrids for Water Purification. ACS Appl. Mater. Interfaces.

[80] Mautner A., Lee K.-Y., Lahtinen P., Hakalahti M., Tammelin T., Li K., Bismarck A. (2014). Nanopapers for organic solvent nanofiltration. Chem. Commun..

[81] Maatar W., Boufi S. (2015). Poly(methacylic acid-co-maleic acid) grafted nanofibrillated cellulose as a reusable novel heavy metal ions adsorbent. Carbohydr. Polym..

[82] Sirviö J.A., Hasa T., Leiviskä T., Liimatainen H., Hormi O. (2016). Bisphosphonate nanocellulose in the removal of vanadium(V) from water. Cellulose.

[83] Abou-Zeid R.E., Dacrory S., Ali K.A., Kamel S. (2018). Novel method of preparation of tricarboxylic cellulose nanofiber for efficient removal of heavy metal ions from aqueous solution. Int. J. Biol. Macromol..

[84] Chen S., Zou Y., Yan Z., Shen W., Shi S., Zhang X., Wang H. (2009). Carboxymethylated-bacterial cellulose for copper and lead ion removal. J. Hazard. Mater..

[85] Jin X., Xiang Z., Liu Q., Chen Y., Lu F. (2017). Polyethyleneimine-bacterial cellulose bioadsorbent for effective removal of copper and lead ions from aqueous solution. Bioresour. Technol..

[86] Oshima T., Kondo K., Ohto K., Inoue K., Baba Y. (2008). Preparation of phosphorylated bacterial cellulose as an adsorbent for metal ions. React. Funct. Polym..

[87] Shen W., Chen S., Shi S., Li X., Zhang X., Hu W., Wang H. (2009). Adsorption of Cu(II) and Pb(II) onto diethylenetriamine-bacterial cellulose. Carbohydr. Polym..

[88] Stephen M., Catherine N., Brenda M., Andrew K., Leslie P., Corrine G. (2011). Oxolane-2,5-dione modified electrospun cellulose nanofibers for heavy metals adsorption. J. Hazard. Mater..

[89] Júnior O.K., Gurgel L.V.A., de Freitas R.P., Gil L.F. (2009). Adsorption of Cu(II), Cd(II), and Pb(II) from aqueous single metal solutions by mercerized cellulose and mercerized sugarcane bagasse chemically modified with EDTA dianhydride (EDTAD). Carbohydr. Polym..

[90] Salama A., Abou-Zeid R.E. (2021). Ionic chitosan/silica nanocomposite as efficient adsorbent for organic dyes. Int. J. Biol. Macromol..

[91] Zhang X., Elsayed I., Navarathna C., Schueneman G.T., Hassan E.B. (2019). Biohybrid Hydrogel and Aerogel from Self-Assembled Nanocellulose and Nanochitin as a High-Efficiency Adsorbent for Water Purification. ACS Appl. Mater. Interfaces.

[92] Pandey S., Do J.Y., Kim J., Kang M. (2020). Fast and highly efficient removal of dye from aqueous solution using natural locust bean gum based hydrogels as adsorbent. Int. J. Biol. Macromol..

[93] Navarro R.R., Sumi K., Matsumura M. (1999). Improved metal affinity of chelating adsorbents through graft polymerization. Water Res..

[94] Hong H.-J., Lim J.S., Hwang J.Y., Kim M., Jeong H.S., Park M.S. (2018). Carboxymethlyated cellulose nanofibrils(CMCNFs) embedded in polyurethane foam as a modular adsorbent of heavy metal ions. Carbohydr. Polym..

[95] Salama A. (2020). Cellulose/silk fibroin assisted calcium phosphate growth: Novel biocomposite for dye adsorption. Int. J. Biol. Macromol..

[96] Abou-Zeid R.E., Salama A., Al-Ahmed Z.A., Awwad N.S., Youssef M.A. (2020). Carboxylated cellulose nanofibers as a novel efficient adsorbent for water purification. Cellul. Chem. Technol..

[97] Manatunga D.C., de Silva R.M., de Silva K.M.N., Ratnaweera R. (2016). Natural polysaccharides leading to super adsorbent hydroxyapatite nanoparticles for the removal of heavy metals and dyes from aqueous solutions. RSC Adv..

[98] Cai J., Lei M., Zhang Q., He J.-R., Chen T., Liu S., Fu S.-H., Li T.-T., Liu G., Fei P. (2017). Electrospun composite nanofiber mats of Cellulose@Organically modified montmorillonite for heavy metal ion removal: Design, characterization, evaluation of absorption performance. Compos. Part A: Appl. Sci. Manuf..

[99] Yao M., Wang Z., Liu Y., Yang G., Chen J. (2019). Preparation of dialdehyde cellulose graftead graphene oxide composite and its adsorption behavior for heavy metals from aqueous solution. Carbohydr. Polym..

[100] Hao Y., Cui Y., Peng J., Zhao N., Li S., Zhai M. (2019). Preparation of graphene oxide/cellulose composites in ionic liquid for Ce (III) removal. Carbohydr. Polym..

[101] Salama A., Hesemann P. (2020). Recent Trends in Elaboration, Processing, and Derivatization of Cellulosic Materials Using Ionic Liquids. ACS Sustain. Chem. Eng..

[102] Ambashta R.D., Sillanpää M. (2010). Water purification using magnetic assistance: A review. J. Hazard. Mater..

[103] Nata I.F., Sureshkumar M., Lee C.-K. (2011). One-pot preparation of amine-rich magnetite/bacterial cellulose nanocomposite and its application for arsenate removal. RSC Adv..

[104] Stoica-Guzun A., Stroescu M., Jinga S.I., Mihalache N., Botez A., Matei C., Berger D., Damian C.M., Ionita V. (2016). Box-Behnken experimental design for chromium(VI) ions removal by bacterial cellulose-magnetite composites. Int. J. Biol. Macromol..

[105] Salama A. (2018). Preparation of CMC-g-P(SPMA) super adsorbent hydrogels: Exploring their capacity for MB removal from waste water. Int. J. Biol. Macromol..

[106] Barhoum A., Favre T., Sayegh S., Tanos F., Coy E., Iatsunskyi I., Razzouk A., Cretin M., Bechelany M. (2021). 3D Self-Supported Nitrogen-Doped Carbon Nanofiber Electrodes Incorporated Co/CoO_x_ Nanoparticles: Application to Dyes Degradation by Electro-Fenton-Based Process. Nanomaterials.

[107] Alila S., Boufi S. (2009). Removal of organic pollutants from water by modified cellulose fibres. Ind. Crop. Prod..

[108] Mautner A., Kobkeatthawin T., Bismarck A. (2017). Efficient continuous removal of nitrates from water with cationic cellulose nanopaper membranes. Resour. Technol..

[109] Bauli C.R., Lima G.F., de Souza A.G., Ferreira R.R., Rosa D.S. (2021). Eco-friendly carboxymethyl cellulose hydrogels filled with nanocellulose or nanoclays for agriculture applications as soil conditioning and nutrient carrier and their impact on cucumber growing. Colloids Surf. A Physicochem. Eng. Asp..

[110] Elfeky A.S., Salem S.S., Elzaref A., Owda M.E., Eladawy H.A., Saeed A., Awad M.A., Abou-Zeid R.E., Fouda A. (2020). Multifunctional cellulose nanocrystal /metal oxide hybrid, photo-degradation, antibacterial and larvicidal activities. Carbohydr. Polym..

[111] Assem Y., Ali K., Abu-Zeid R., Kame S. (2019). Synthesis of Acrylate-modified Cellulose via RAFT Polymerization and its Application as Efficient Metal Ions Adsorbent. Egypt. J. Chem..

[112] Salama A., Shukry N., El-Sakhawy M. (2015). Carboxymethyl cellulose-g-poly(2-(dimethylamino) ethyl methacrylate) hydrogel as adsorbent for dye removal. Int. J. Biol. Macromol..

[113] Salama A., Shoueir K.R., Aljohani H.A. (2017). Preparation of sustainable nanocomposite as new adsorbent for dyes removal. Fibers Polym..

[114] Salama A., Aljohani H.A., Shoueir K.R. (2018). Oxidized cellulose reinforced silica gel: New hybrid for dye adsorption. Mater. Lett..

[115] Abou-Zeid R.E., Awwad N.S., Nabil S., Salama A., Youssef M.A. (2019). Oxidized alginate/gelatin decorated silver nanoparticles as new nanocomposite for dye adsorption. Int. J. Biol. Macromol..

[116] Varjani S.J., Gnansounou E., Pandey A. (2017). Comprehensive review on toxicity of persistent organic pollutants from petroleum refinery waste and their degradation by microorganisms. Chemosphere.

[117] Owda M.E., Elfeky A.S., Abouzeid R.E., Saleh A.K., Awad M.A., Abdellatif H.A., Ahmed F.M., Elzaref A.S. (2021). Enhancement of photocatalytic and biological activities of chitosan/activated carbon incorporated with TiO_2_ nanoparticles. Environ. Sci. Pollut. Res..

[118] Qiao H., Zhou Y., Yu F., Wang E., Min Y., Huang Q., Pang L., Ma T. (2015). Effective removal of cationic dyes using carboxylate-functionalized cellulose nanocrystals. Chemosphere.

[119] Salama A. (2017). New sustainable hybrid material as adsorbent for dye removal from aqueous solutions. J. Colloid Interface Sci..

[120] Salama A., Etri S., Mohamed S.A., El-Sakhawy M. (2018). Carboxymethyl cellulose prepared from mesquite tree: New source for promising nanocomposite materials. Carbohydr. Polym..

[121] Salama A. (2016). Functionalized hybrid materials assisted organic dyes removal from aqueous solutions. Environ. Nanotechnol. Monit. Manag..

[122] Mousa H.M., Fahmy H.S., Abouzeid R., Abdel-Jaber G., Ali W. (2022). Polyvinylidene fluoride-cellulose nanocrystals hybrid nanofiber membrane for energy harvesting and oil-water separation applications. Mater. Lett..

[123] Abou-Zeid R.E., Ali K.A., Gawad R.M.A., Kamal K.H., Kamel S., Khiari R. (2021). Removal of Cu(II), Pb(II), Mg(II), and Fe(II) by Adsorption onto Alginate/Nanocellulose Beads as Bio-Sorbent. J. Renew. Mater..

[124] Shahnaz T., Priyan V.V., Pandian S., Narayanasamy S. (2021). Use of Nanocellulose extracted from grass for adsorption abatement of Ciprofloxacin and Diclofenac removal with phyto, and fish toxicity studies. Environ. Pollut..

[125] Li M., Li X., Wang L., Pei Y., An M., Liu J., Zheng X., Tang K. (2021). Highly efficient and selective removal of anionic dyes from water using a cellulose nanofibril/chitosan sponge prepared by dehydrothermal treatment. J. Environ. Chem. Eng..

[126] Yu Z., Hu C., DiChiara A.B., Jiang W., Gu J. (2020). Cellulose Nanofibril/Carbon Nanomaterial Hybrid Aerogels for Adsorption Removal of Cationic and Anionic Organic Dyes. Nanomaterials.

[127] Batmaz R., Mohammed N., Zaman M., Minhas G., Berry R.M., Tam K.C. (2014). Cellulose nanocrystals as promising adsorbents for the removal of cationic dyes. Cellulose.

[128] Yu H., Zhang D.-Z., Lu F.-F., Yao J. (2016). New Approach for Single-Step Extraction of Carboxylated Cellulose Nanocrystals for Their Use as Adsorbents and Flocculants. ACS Sustain. Chem. Eng..

[129] He X., Male K.B., Nesterenko P.N., Brabazon D., Paull B., Luong J.H. (2013). Adsorption and Desorption of Methylene Blue on Porous Carbon Monoliths and Nanocrystalline Cellulose. ACS Appl. Mater. Interfaces.

[130] Jin L., Sun Q., Xu Q., Xu Y. (2015). Adsorptive removal of anionic dyes from aqueous solutions using microgel based on nanocellulose and polyvinylamine. Bioresour. Technol..

[131] Voisin H., Bergström L., Liu P., Mathew A.P. (2017). Nanocellulose-based materials for water purification. Nanomaterials.

[132] Ma H., Burger C., Hsiao B.S., Chu B. (2012). Nanofibrous Microfiltration Membrane Based on Cellulose Nanowhiskers. Biomacromolecules.

[133] Song K., Xu H., Xu L., Xie K., Yang Y. (2017). Cellulose nanocrystal-reinforced keratin bioadsorbent for effective removal of dyes from aqueous solution. Bioresour. Technol..

[134] Mohammed N., Grishkewich N., Berry R.M., Tam K.C. (2015). Cellulose nanocrystal–alginate hydrogel beads as novel adsorbents for organic dyes in aqueous solutions. Cellulose.

[135] Zhou C., Lee S., Dooley K., Wu Q. (2013). A facile approach to fabricate porous nanocomposite gels based on partially hydrolyzed polyacrylamide and cellulose nanocrystals for adsorbing methylene blue at low concentrations. J. Hazard. Mater..

[136] Melo B.C., Paulino F.A., Cardoso V.A., Pereira A.G., Fajardo A.R., Rodrigues F.H. (2018). Cellulose nanowhiskers improve the methylene blue adsorption capacity of chitosan-g-poly(acrylic acid) hydrogel. Carbohydr. Polym..

[137] Eyley S., Thielemans W. (2011). Imidazolium grafted cellulose nanocrystals for ion exchange applications. Chem. Commun..

[138] Chan C.H., Chia C.H., Zakaria S., Sajab M.S., Chin S.X. (2015). Cellulose nanofibrils: A rapid adsorbent for the removal of methylene blue. RSC Adv..

[139] Pei A., Butchosa N., Berglund L.A., Zhou Q. (2013). Surface quaternized cellulose nanofibrils with high water absorbency and adsorption capacity for anionic dyes. Soft Matter.

[140] Salama A. (2019). Soy protein acid hydrolysate/silica hybrid material as novel adsorbent for methylene blue. Compos. Commun..

[141] Salama A. (2019). Cellulose/calcium phosphate hybrids: New materials for biomedical and environmental applications. Int. J. Biol. Macromol..

[142] Lu M., Li G., Yang Y., Yu Y. (2021). A review on in-vitro oral bioaccessibility of organic pollutants and its application in human exposure assessment. Sci. Total Environ..

[143] Yang J., Ma C., Tao J., Li J., Du K., Wei Z., Chen C., Wang Z., Zhao C., Ma M. (2020). Optimization of polyvinylamine-modified nanocellulose for chlorpyrifos adsorption by central composite design. Carbohydr. Polym..

[144] Khalaf B., Hamed O., Jodeh S., Hanbali G., Bol R., Dagdag O., Samhan S. (2021). Novel, Environment-Friendly Cellulose-Based Derivatives for Tetraconazole Removal from Aqueous Solution. Polymers.

[145] Ma C., Yi L., Yang J., Tao J., Li J. (2020). Nanocellulose–organic montmorillonite nanocomposite adsorbent for diuron removal from aqueous solution: Optimization using response surface methodology. RSC Adv..

[146] Nikou M., Samadi-Maybodi A., Yasrebi K., Sedighi-Pashaki E. (2021). Simultaneous monitoring of the adsorption process of two organophosphorus pesticides by employing GO/ZIF-8 composite as an adsorbent. Environ. Technol. Innov..

[147] Zhou Q., Wang W., Liu F., Chen R. (2021). Removal of difenoconazole and nitenpyram by composite calcium alginate beads during apple juice clarification. Chemosphere.

[148] Meneguin A., Pacheco G., Silva J.M., de Araujo F.P., Silva-Filho E.C., Bertolino L.C., Barud H.D.S. (2021). Nanocellulose/palygorskite biocomposite membranes for controlled release of metronidazole. Int. J. Biol. Macromol..

[149] Hosseinzadeh S., Hosseinzadeh H., Pashaei S. (2019). Fabrication of nanocellulose loaded poly(AA-*co*-HEMA) hydrogels for ceftriaxone controlled delivery and crystal violet adsorption. Polym. Compos..

[150] Zhu Q., Wang Y., Li M., Liu K., Hu C., Yan K., Sun G., Wang D. (2017). Activable carboxylic acid functionalized crystalline nanocellulose/PVA-*co*-PE composite nanofibrous membrane with enhanced adsorption for heavy metal ions. Sep. Purif. Technol..

[151] Taleb K., Markovski J., Veličković Z., Rusmirović J., Rancic M., Pavlović V., Marinković A. (2019). Arsenic removal by magnetite-loaded amino modified nano/microcellulose adsorbents: Effect of functionalization and media size. Arab. J. Chem..

[152] Wang S., Ma X., Zheng P. (2019). Sulfo-functional 3D porous cellulose/graphene oxide composites for highly efficient removal of methylene blue and tetracycline from water. Int. J. Biol. Macromol..

[153] Sehaqui H., Mautner A., de Larraya U.P., Pfenninger N., Tingaut P., Zimmermann T. (2016). Cationic cellulose nanofibers from waste pulp residues and their nitrate, fluoride, sulphate and phosphate adsorption properties. Carbohydr. Polym..

[154] Rethinasabapathy M., Kang S.-M., Lee I., Lee G.-W., Hwang S.K., Roh C., Huh Y.S. (2018). Layer-Structured POSS-Modified Fe-Aminoclay/Carboxymethyl Cellulose Composite as a Superior Adsorbent for the Removal of Radioactive Cesium and Cationic Dyes. Ind. Eng. Chem. Res..

[155] Said M.M., Rehan M., El-Sheikh S.M., Zahran M.K., Abdel-Aziz M.S., Bechelany M., Barhoum A. (2021). Multifunctional Hydroxyapatite/Silver Nanoparticles/Cotton Gauze for Antimicrobial and Biomedical Applications. Nanomaterials.

[156] Valencia L., Kumar S., Nomena E.M., Salazar-Alvarez G., Mathew A.P. (2020). In-Situ Growth of Metal Oxide Nanoparticles on Cellulose Nanofibrils for Dye Removal and Antimicrobial Applications. ACS Appl. Nano Mater..

[157] Hassan H.S., Elkady M., Farghali A., Salem A.M., El-Hamid A.A. (2017). Fabrication of novel magnetic zinc oxide cellulose acetate hybrid nano-fiber to be utilized for phenol decontamination. J. Taiwan Inst. Chem. Eng..

[158] Chen L., Berry R.M., Tam K.C. (2014). Synthesis of β-Cyclodextrin-Modified Cellulose Nanocrystals (CNCs)@Fe_3_O_4_@SiO_2_ Superparamagnetic Nanorods. ACS Sustain. Chem. Eng..

[159] Maatar W., Alila S., Boufi S. (2013). Cellulose based organogel as an adsorbent for dissolved organic compounds. Ind. Crop. Prod..

[160] Yousefi N., Wong K.K.W., Hosseinidoust Z., Sørensen H.O., Bruns S., Zheng Y., Tufenkji N. (2018). Hierarchically porous, ultra-strong reduced graphene oxide-cellulose nanocrystal sponges for exceptional adsorption of water contaminants. Nanoscale.

[161] Chen L., Li Y., Hu S., Sun J., Du Q., Yang X., Ji Q., Wang Z., Wang D., Xia Y. (2016). Removal of methylene blue from water by cellulose/graphene oxide fibres. J. Exp. Nanosci..

[162] Bhattacharyya S., Klerks P., Nyman J. (2003). Toxicity to freshwater organisms from oils and oil spill chemical treatments in laboratory microcosms. Environ. Pollut..

[163] Wang W., Lin J., Cheng J., Cui Z., Si J., Wang Q., Peng X., Turng L.-S. (2020). Dual super-amphiphilic modified cellulose acetate nanofiber membranes with highly efficient oil/water separation and excellent antifouling properties. J. Hazard. Mater..

[164] Zhang Z., Sèbe G., Rentsch D., Zimmermann T., Tingaut P. (2014). Ultralightweight and Flexible Silylated Nanocellulose Sponges for the Selective Removal of Oil from Water. Chem. Mater..

[165] Sharma P.R., Sharma S.K., Lindström T., Hsiao B.S. (2020). Nanocellulose-Enabled Membranes for Water Purification: Perspectives. Adv. Sustain. Syst..

[166] Zanini M., Lavoratti A., Lazzari L.K., Galiotto D., Pagnocelli M., Baldasso C., Zattera A.J. (2017). Producing aerogels from silanized cellulose nanofiber suspension. Cellulose.

[167] Korhonen J.T., Hiekkataipale P., Malm J., Karppinen M., Ikkala O., Ras R.H.A. (2011). Inorganic Hollow Nanotube Aerogels by Atomic Layer Deposition onto Native Nanocellulose Templates. ACS Nano.

[168] Meng Y., Young T.M., Liu P., Contescu C.I., Huang B., Wang S. (2015). Ultralight carbon aerogel from nanocellulose as a highly selective oil absorption material. Cellulose.

[169] Sai H., Fu R., Xing L., Xiang J., Li Z., Li F., Zhang T. (2015). Surface Modification of Bacterial Cellulose Aerogels’ Web-like Skeleton for Oil/Water Separation. ACS Appl. Mater. Interfaces.

[170] Jeddi M.K., Laitinen O., Liimatainen H. (2019). Magnetic superabsorbents based on nanocellulose aerobeads for selective removal of oils and organic solvents. Mater. Des..

[171] Gu H., Zhou X., Lyu S., Pan D., Dong M., Wu S., Ding T., Wei X., Seok I., Wei S. (2020). Magnetic nanocellulose-magnetite aerogel for easy oil adsorption. J. Colloid Interface Sci..

[172] Grishkewich N., Mohammed N., Tang J., Tam K.C. (2017). Recent advances in the application of cellulose nanocrystals. Curr. Opin. Colloid Interface Sci..

[173] Lee C.S., Robinson J., Chong M.F. (2014). A review on application of flocculants in wastewater treatment. Process. Saf. Environ. Prot..

[174] Suopajärvi T., Liimatainen H., Hormi O., Niinimäki J. (2013). Coagulation–flocculation treatment of municipal wastewater based on anionized nanocelluloses. Chem. Eng. J..

[175] Korhonen M., Laine J. (2014). Flocculation and retention of fillers with nanocelluloses. Nord. Pulp Pap. Res. J..

[176] Zhang L.-W., Hua J.-R., Zhu W.-J., Liu L., Du X.-L., Meng R.-J., Yao J.-M. (2018). Flocculation Performance of Hyperbranched Polyethylenimine-Grafted Cellulose in Wastewater Treatment. ACS Sustain. Chem. Eng..

[177] Kemppainen K., Suopajärvi T., Laitinen O., Ämmälä A., Liimatainen H., Illikainen M. (2016). Flocculation of fine hematite and quartz suspensions with anionic cellulose nanofibers. Chem. Eng. Sci..

[178] Campano C., Lopez-Exposito P., Blanco A., Negro C., van de Ven T.G. (2019). Hairy cationic nanocrystalline cellulose as a novel flocculant of clay. J. Colloid Interface Sci..

[179] Sun X., Danumah C., Liu Y., Boluk Y. (2012). Flocculation of bacteria by depletion interactions due to rod-shaped cellulose nanocrystals. Chem. Eng. J..

[180] Vandamme D., Eyley S., Van den Mooter G., Muylaert K., Thielemans W. (2015). Highly charged cellulose-based nanocrystals as flocculants for harvesting Chlorella vulgaris. Bioresour. Technol..

[181] Akhlaghi S.P., Zaman M., Mohammed N., Brinatti C., Batmaz R., Berry R., Loh W., Tam K.C. (2015). Synthesis of amine functionalized cellulose nanocrystals: Optimization and characterization. Carbohydr. Res..

[182] Suopajärvi T., Koivuranta E., Liimatainen H., Niinimäki J. (2014). Flocculation of municipal wastewaters with anionic nanocelluloses: Influence of nanocellulose characteristics on floc morphology and strength. J. Environ. Chem. Eng..

[183] Quinlan P.J., Tanvir A., Tam K.C. (2015). Application of the central composite design to study the flocculation of an anionic azo dye using quaternized cellulose nanofibrils. Carbohydr. Polym..

[184] Zheng W.-L., Hu W.-L., Chen S.-Y., Zheng Y., Zhou B.-H., Wang H.-P. (2014). High photocatalytic properties of zinc oxide nanoparticles with amidoximated bacterial cellulose nanofibers as templates. Chin. J. Polym. Sci..

[185] Wang S.-D., Ma Q., Liu H., Wang K., Ling L.-Z., Zhang K.-Q. (2015). Robust electrospinning cellulose acetate@TiO_2_ ultrafine fibers for dyeing water treatment by photocatalytic reactions. RSC Adv..

[186] Rehan M., Barhoum A., Khattab T., Gätjen L., Wilken R. (2019). Colored, photocatalytic, antimicrobial and UV-protected viscose fibers decorated with Ag/Ag_2_CO_3_ and Ag/Ag_3_PO_4_ nanoparticles. Cellulose.

[187] Leong W.S., Luo X., Li Y., Khoo K.H., Quek S.Y., Thong J.T.L. (2015). Low Resistance Metal Contacts to MoS_2_ Devices with Nickel-Etched-Graphene Electrodes. ACS Nano.

[188] Rehan M., Barhoum A., Van Assche G., Dufresne A., Gätjen L., Wilken R. (2017). Towards multifunctional cellulosic fabric: UV photo-reduction and in-situ synthesis of silver nanoparticles into cellulose fabrics. Int. J. Biol. Macromol..

[189] Barhoum A., Melcher J., Van Assche G., Rahier H., Bechelany M., Fleisch M., Bahnemann D.B.D. (2017). Synthesis, growth mechanism, and photocatalytic activity of Zinc oxide nanostructures: Porous microparticles versus nonporous nanoparticles. J. Mater. Sci..

[190] Ren C., Yang B., Wu M., Xu J., Fu Z., Lv Y., Guo T., Zhao Y., Zhu C. (2010). Synthesis of Ag/ZnO nanorods array with enhanced photocatalytic performance. J. Hazard. Mater..

[191] Lin Z., Lu Y., Huang J. (2019). A hierarchical Ag_2_O-nanoparticle/TiO_2_-nanotube composite derived from natural cellulose substance with enhanced photocatalytic performance. Cellulose.

[192] Ali S., Rehman S.A.U., Luan H.-Y., Farid M.U., Huang H. (2019). Challenges and opportunities in functional carbon nanotubes for membrane-based water treatment and desalination. Sci. Total Environ..

[193] Chang H.N., Hou S.X., Hao Z.C., Cui G.H. (2018). Ultrasonic green synthesis of an Ag/CP nanocomposite for enhanced photodegradation effectiveness. Ultrason. Sonochem..

[194] Gan L., Geng A., Xu L., Chen M., Wang L., Liu J., Han S., Mei C., Zhong Q. (2018). The fabrication of bio-renewable and recyclable cellulose based carbon microspheres incorporated by CoFe2O4 and the photocatalytic properties. J. Clean. Prod..

[195] Zeng J., Liu S., Cai J., Zhang L. (2010). TiO2 Immobilized in Cellulose Matrix for Photocatalytic Degradation of Phenol under Weak UV Light Irradiation. J. Phys. Chem. C.

[196] Jiao Y., Wan C., Bao W., Gao H., Liang D., Li J. (2018). Facile hydrothermal synthesis of Fe_3_O_4_@cellulose aerogel nanocomposite and its application in Fenton-like degradation of Rhodamine B. Carbohydr. Polym..

[197] Yanfen F., Yingping H., Jing Y., Pan W., Genwei C. (2011). Unique ability of BiOBr to decarboxylate D-Glu and D-MeAsp in the photocatalytic degradation of microcystin-LR in water. Environ. Sci. Technol..

[198] Li Q., Zhou D., Zhang P., Man P., Tian Z., Li Y., Ai S. (2016). The BiOBr/regenerated cellulose composite film as a green catalyst for light degradation of phenol. Colloids Surf. A Physicochem. Eng. Asp..

[199] Gaur M., Misra C., Yadav A.B., Swaroop S., Maolmhuaidh F.Ó., Bechelany M., Barhoum A. (2021). Biomedical Applications of Carbon Nanomaterials: Fullerenes, Quantum Dots, Nanotubes, Nanofibers, and Graphene. Materials.

[200] Wang Z., Ma H., Chu B., Hsiao B.S. (2017). Fabrication of cellulose nanofiber-based ultrafiltration membranes by spray coating approach. J. Appl. Polym. Sci..

[201] Azzaoui K., Mejdoubi E., Lamhamdi A., Jodeh S., Hamed O., Berrabah M., Jerdioui S., Salghi R., Akartasse N., Errich A. (2017). Preparation and characterization of biodegradable nanocomposites derived from carboxymethyl cellulose and hydroxyapatite. Carbohydr. Polym..

[202] Peng N., Hu D., Zeng J., Li Y., Liang L., Chang C. (2016). Superabsorbent Cellulose–Clay Nanocomposite Hydrogels for Highly Efficient Removal of Dye in Water. ACS Sustain. Chem. Eng..

[203] Yadav M., Rhee K.Y., Park S.-J. (2014). Synthesis and characterization of graphene oxide/carboxymethylcellulose/alginate composite blend films. Carbohydr. Polym..

[204] Wei X., Huang T., Yang J.-H., Zhang N., Wang Y., Zhou Z.-W. (2017). Green synthesis of hybrid graphene oxide/microcrystalline cellulose aerogels and their use as superabsorbents. J. Hazard. Mater..

[205] Fang Q., Zhou X., Deng W., Zheng Z., Liu Z. (2016). Freestanding bacterial cellulose-graphene oxide composite membranes with high mechanical strength for selective ion permeation. Sci. Rep..

[206] Deng C., Liu J., Zhou W., Zhang Y.-K., Du K.-F., Zhao Z.-M. (2012). Fabrication of spherical cellulose/carbon tubes hybrid adsorbent anchored with welan gum polysaccharide and its potential in adsorbing methylene blue. Chem. Eng. J..

[207] Qin Y., Qin Z., Liu Y., Cheng M., Qian P., Wang Q., Zhu M. (2015). Superparamagnetic iron oxide coated on the surface of cellulose nanospheres for the rapid removal of textile dye under mild condition. Appl. Surf. Sci..

[208] Fallah M.H., Fallah S.A., Zanjanchi M.A. (2011). Synthesis and Characterization of Nano-sized Zinc Oxide Coating on Cellulosic Fibers: Photoactivity and Flame-retardancy Study. Chin. J. Chem..

[209] Ali K., Dwivedi S., Azam A., Saquib Q., Al-Said M.S., Al-Khedhairy A., Musarrat J. (2016). Aloe vera extract functionalized zinc oxide nanoparticles as nanoantibiotics against multi-drug resistant clinical bacterial isolates. J. Colloid Interface Sci..

[210] Hamad H., Bailón-García E., Morales-Torres S., Carrasco-Marín F., Pérez-Cadenas A., Maldonado-Hódar F.J. (2018). Physicochemical properties of new cellulose-TiO_2_ composites for the removal of water pollutants: Developing specific interactions and performances by cellulose functionalization. J. Environ. Chem. Eng..

[211] Nsib M.F., Hajji F., Mayoufi A., Moussa N., Rayes A., Houas A. (2014). In situ synthesis and characterization of TiO2/HPM cellulose hybrid material for the photocatalytic degradation of 4-NP under visible light. Comptes Rendus Chim..

[212] Shoukat A., Wahid F., Khan T., Siddique M., Nasreen S., Yang G., Ullah M.W., Khan R. (2019). Titanium oxide-bacterial cellulose bioadsorbent for the removal of lead ions from aqueous solution. Int. J. Biol. Macromol..

[213] Tanzifi M., Yaraki M.T., Karami M., Karimi S., Kiadehi A.D., Karimipour K., Wang S. (2018). Modelling of dye adsorption from aqueous solution on polyaniline/carboxymethyl cellulose/TiO_2_ nanocomposites. J. Colloid Interface Sci..

[214] Matsubara H., Takada M., Koyama S., Hashimoto K., Fujishima A. (1995). Photoactive TiO_2_ Containing Paper: Preparation and Its Photocatalytic Activity under Weak UV Light Illumination. Chem. Lett..

[215] Suman, Kardam A., Gera M., Jain V. (2015). A novel reusable nanocomposite for complete removal of dyes, heavy metals and microbial load from water based on nanocellulose and silver nano-embedded pebbles. Environ. Technol..

[216] Patel D.K., Dutta S.D., Lim K.-T. (2019). Nanocellulose-based polymer hybrids and their emerging applications in biomedical engineering and water purification. RSC Adv..

[217] Patil K., Jeong S., Lim H., Byun H.-S., Han S. (2019). Removal of volatile organic compounds from air using activated carbon impregnated cellulose acetate electrospun mats. Environ. Eng. Res..

[218] Samyn P., Barhoum A. (2018). Engineered nanomaterials for papermaking industry. Fundamentals of Nanoparticles.

[219] Tavakolian M., Jafari S.M., van de Ven T.G.M. (2020). A Review on Surface-Functionalized Cellulosic Nanostructures as Biocompatible Antibacterial Materials. Nano-Micro Lett..

[220] Wang Z., Zhang W., Yu J., Zhang L., Liu L., Zhou X., Huang C., Fan Y. (2019). Preparation of nanocellulose/filter paper (NC/FP) composite membranes for high-performance filtration. Cellulose.

[221] Zhao J., Lu Z., He X., Zhang X., Li Q., Xia T., Zhang W., Lu C., Deng Y. (2017). One-Step Fabrication of Fe(OH)_3_@Cellulose Hollow Nanofibers with Superior Capability for Water Purification. ACS Appl. Mater. Interfaces.

[222] Yang R., Aubrecht K.B., Ma H., Wang R., Grubbs R.B., Hsiao B.S., Chu B. (2014). Thiol-modified cellulose nanofibrous composite membranes for chromium (VI) and lead (II) adsorption. Polymer.

[223] Wang X., Ding B., Sun G., Wang M., Yu J. (2013). Electro-spinning/netting: A strategy for the fabrication of three-dimensional polymer nano-fiber/nets. Prog. Mater. Sci..

[224] Mansouri J., Harrisson S., Chen V. (2010). Strategies for controlling biofouling in membrane filtration systems: Challenges and opportunities. J. Mater. Chem..

[225] Kong L., Yin X., Yuan X., Zhang Y., Liu X., Cheng L., Zhang L. (2014). Electromagnetic wave absorption properties of graphene modified with carbon nanotube/poly(dimethyl siloxane) composites. Carbon.

[226] Hassan M., Berglund L., Abou-Zeid R., Hassan E., Abou-Elseoud W., Oksman K. (2019). Nanocomposite Film Based on Cellulose Acetate and Lignin-Rich Rice Straw Nanofibers. Materials.

[227] Xu X., Zhou J., Jiang L., Lubineau G., Chen Y., Wu X.-F., Piere R. (2013). Porous core-shell carbon fibers derived from lignin and cellulose nanofibrils. Mater. Lett..

[228] Ma H., Burger C., Hsiao B.S., Chu B. (2012). Highly Permeable Polymer Membranes Containing Directed Channels for Water Purification. ACS Macro Lett..

[229] Wang J.-G., Yang Y., Huang Z.-H., Kang F. (2013). A high-performance asymmetric supercapacitor based on carbon and carbon–MnO_2_ nanofiber electrodes. Carbon.

[230] Ferraz E.R.A., Oliveira G.A.R., Grando M.D., Lizier T.M., Zanoni M.V.B., Oliveira D.P. (2013). Photoelectrocatalysis based on Ti/TiO_2_ nanotubes removes toxic properties of the azo dyes Disperse Red 1, Disperse Red 13 and Disperse Orange 1 from aqueous chloride samples. J. Environ. Manag..

[231] Barud H.S., Souza J.L., Santos D.B., Crespi M.S., Ribeiro C., Messaddeq Y., Ribeiro S. (2011). Bacterial cellulose/poly(3-hydroxybutyrate) composite membranes. Carbohydr. Polym..

[232] Asper M., Hanrieder T., Quellmalz A., Mihranyan A. (2015). Removal of xenotropic murine leukemia virus by nanocellulose based filter paper. Biologicals.

[233] Metreveli G., Wågberg L., Emmoth E., Belák S., Strømme M., Mihranyan A. (2014). A Size-Exclusion Nanocellulose Filter Paper for Virus Removal. Adv. Healthc. Mater..

[234] Quellmalz A., Mihranyan A. (2015). Citric Acid Cross-Linked Nanocellulose-Based Paper for Size-Exclusion Nanofiltration. ACS Biomater. Sci. Eng..

[235] Song Y., Seo J.Y., Kim H., Beak K.Y. (2019). Structural control of cellulose nanofibrous composite membrane with metal organic framework (ZIF-8) for highly selective removal of cationic dye. Carbohydr. Polym..

[236] Takai M., Nonomura F., Inukai T., Fujiwara M., Hayashi J. (1991). Filtration and permeation characteristics of bacterial cellulose composite. Sen’i Gakkaishi.

[237] Mautner A., Lee K.-Y., Tammelin T., Mathew A.P., Nedoma A., Li K., Bismarck A. (2015). Cellulose nanopapers as tight aqueous ultra-filtration membranes. React. Funct. Polym..

[238] Cheng Q., Ye D., Chang C., Zhang L. (2017). Facile fabrication of superhydrophilic membranes consisted of fibrous tunicate cellulose nanocrystals for highly efficient oil/water separation. J. Membr. Sci..

[239] Karim Z., Claudpierre S., Grahn M., Oksman K., Mathew A.P. (2016). Nanocellulose based functional membranes for water cleaning: Tailoring of mechanical properties, porosity and metal ion capture. J. Membr. Sci..

[240] Karim Z., Mathew A.P., Kokol V., Wei J., Grahn M. (2016). High-flux affinity membranes based on cellulose nanocomposites for removal of heavy metal ions from industrial effluents. RSC Adv..

[241] Karim Z., Hakalahti M., Tammelin T., Mathew A.P. (2017). In situ TEMPO surface functionalization of nanocellulose membranes for enhanced adsorption of metal ions from aqueous medium. RSC Adv..

[242] Karim Z., Mathew A.P., Grahn M., Mouzon J., Oksman K. (2014). Nanoporous membranes with cellulose nanocrystals as functional entity in chitosan: Removal of dyes from water. Carbohydr. Polym..

[243] Goetz L.A., Naseri N., Nair S.S., Karim Z., Mathew A.P. (2018). All cellulose electrospun water purification membranes nanotextured using cellulose nanocrystals. Cellulose.

[244] Soyekwo F., Zhang Q.G., Lin X.C., Wu X.M., Zhu A.M., Liu Q.L. (2016). Facile preparation and separation performances of cellulose nanofibrous membranes. J. Appl. Polym. Sci..

[245] Mautner A., Maples H.A., Kobkeatthawin T., Kokol V., Karim Z., Li K., Bismarck A. (2016). Phosphorylated nanocellulose papers for copper adsorption from aqueous solutions. Int. J. Environ. Sci. Technol..

[246] Varanasi S., Low Z.-X., Batchelor W. (2015). Cellulose nanofibre composite membranes—Biodegradable and recyclable UF membranes. Chem. Eng. J..

[247] Wang R., Guan S., Sato A., Wang X., Wang Z., Yang R., Hsiao B.S., Chu B. (2013). Nanofibrous microfiltration membranes capable of removing bacteria, viruses and heavy metal ions. J. Membr. Sci..

[248] Xiong Y., Wang C., Wang H., Jin C., Sun Q., Xu X. (2018). Nano-cellulose hydrogel coated flexible titanate-bismuth oxide membrane for trinity synergistic treatment of super-intricate anion/cation/oily-water. Chem. Eng. J..

[249] Wanichapichart P., Kaewnopparat S., Buaking K., Puthai W. (2002). Characterization of cellulose membranes produced by Acetobacter xyllinum. Songklanakarin J. Sci. Technol..

[250] Chen S., Teng Q. (2017). Quantitative Immobilization of Phthalocyanine onto Bacterial Cellulose for Construction of a High-Performance Catalytic Membrane Reactor. Materials.

[251] Hossain F., Perales-Perez O.J., Hwang S., Román F. (2014). Antimicrobial nanomaterials as water disinfectant: Applications, limitations and future perspectives. Sci. Total Environ..

[252] Li J., Cha R., Mou K., Zhao X., Long K., Luo H., Zhou F., Jiang X. (2018). Nanocellulose-Based Antibacterial Materials. Adv. Healthc. Mater..

[253] Abitbol T., Marway H., Cranston E.D. (2014). Surface modification of cellulose nanocrystals with cetyltrimethylammonium bromide. Nord. Pulp Pap. Res. J..

[254] Fei P., Liao L., Meng J., Cheng B., Hu X., Song J. (2018). Non-leaching antibacterial cellulose triacetate reverse osmosis membrane via covalent immobilization of quaternary ammonium cations. Carbohydr. Polym..

[255] Yao J., Fang W., Guo J., Jiao D., Chen S., Ifuku S., Wang H., Walther A. (2020). Highly Mineralized Biomimetic Polysaccharide Nanofiber Materials Using Enzymatic Mineralization. Biomacromolecules.

[256] Li M., Liu X., Liu N., Guo Z., Singh P.K., Fu S. (2018). Effect of surface wettability on the antibacterial activity of nanocellulose-based material with quaternary ammonium groups. Colloids Surf. A Physicochem. Eng. Asp..

[257] Fu F., Gu J., Cao J., Shen R., Liu H., Zhang Y., Liu X., Zhou J. (2018). Reduction of Silver Ions Using an Alkaline Cellulose Dope: Straightforward Access to Ag/ZnO Decorated Cellulose Nanocomposite Film with Enhanced Antibacterial Activities. ACS Sustain. Chem. Eng..

[258] Nguyen H.-L., Jo Y.K., Cha M., Cha Y.J., Yoon D.K., Sanandiya N.D., Prajatelistia E., Oh D.X., Hwang D.S. (2016). Mussel-Inspired Anisotropic Nanocellulose and Silver Nanoparticle Composite with Improved Mechanical Properties, Electrical Conductivity and Antibacterial Activity. Polymers.

[259] Carpenter A.W., De Lannoy C.-F., Wiesner M.R. (2015). Cellulose Nanomaterials in Water Treatment Technologies. Environ. Sci. Technol..

[260] Salama A., Neumann M., Günter C., Taubert A. (2014). Ionic liquid-assisted formation of cellulose/calcium phosphate hybrid materials. Beilstein J. Nanotechnol..

[261] Salama A. (2017). Dicarboxylic cellulose decorated with silver nanoparticles as sustainable antibacterial nanocomposite material. Environ. Nanotechnol. Monit. Manag..

